# Protein Complexes and Proteolytic Activation of the Cell Wall Hydrolase RipA Regulate Septal Resolution in Mycobacteria

**DOI:** 10.1371/journal.ppat.1003197

**Published:** 2013-02-28

**Authors:** Michael C. Chao, Karen J. Kieser, Shoko Minami, Daniela Mavrici, Bree B. Aldridge, Sarah M. Fortune, Tom Alber, Eric J. Rubin

**Affiliations:** 1 Department of Immunology and Infectious Diseases, Harvard School of Public Health, Boston, Massachusetts, United States of America; 2 Department of Molecular and Cell Biology, QB3 Institute, University of California-Berkeley, Berkeley, California, United States of America; University of Alabama at Birmingham, United States of America

## Abstract

Peptidoglycan hydrolases are a double-edged sword. They are required for normal cell division, but when dysregulated can become autolysins lethal to bacteria. How bacteria ensure that peptidoglycan hydrolases function only in the correct spatial and temporal context remains largely unknown. Here, we demonstrate that dysregulation converts the essential mycobacterial peptidoglycan hydrolase RipA to an autolysin that compromises cellular structural integrity. We find that mycobacteria control RipA activity through two interconnected levels of regulation *in vivo*—protein interactions coordinate PG hydrolysis, while proteolysis is necessary for RipA enzymatic activity. Dysregulation of RipA protein complexes by treatment with a peptidoglycan synthase inhibitor leads to excessive RipA activity and impairment of correct morphology. Furthermore, expression of a RipA dominant negative mutant or of differentially processed RipA homologues reveals that RipA is produced as a zymogen, requiring proteolytic processing for activity. The amount of RipA processing differs between fast-growing and slow-growing mycobacteria and correlates with the requirement for peptidoglycan hydrolase activity in these species. Together, the complex picture of RipA regulation is a part of a growing paradigm for careful control of cell wall hydrolysis by bacteria during growth, and may represent a novel target for chemotherapy development.

## Author Summary

Peptidoglycan (PG) is a major component of the bacterial cell wall, which forms a flexible, but strong mesh around the cell to oppose osmotic pressure and prevent lysis. PG is also dynamically modified, continually being disassembled and polymerized as the cell elongates and divides. It remains poorly understood how cells can titrate enough hydrolysis of the PG to allow bacterial growth without leading to excessive digestion and disruption of cellular integrity. In our work, we have identified two methods by which a critical PG hydrolase, RipA, is carefully controlled in *Mycobacterium tuberculosis*—protein interactions help prevent lethal RipA dysregulation, while proteolytic cleavage is used as a second step to activate the enzyme in order to separate daughter cells. Our work elaborates multiple post-transcriptional mechanisms for preventing PG hydrolases from becoming lethal autolysins. These different levels of regulation may serve as a more general paradigm for PG remodeling in other bacterial species.

## Introduction

*Mycobacterium tuberculosis* is the causative agent of tuberculosis and accounts for up to 10 million symptomatic infections a year [1]. The spread of multi-, extensively- and now totally- drug resistant strains [2] has created a pressing need to understand essential mycobacterial processes in an effort to define novel targets for chemotherapy. One highly essential bacterial process is peptidoglycan (PG) synthesis and remodeling, which is critical for providing structural integrity in nearly all bacteria. PG forms a continuous macromolecular mesh that is part of the bacterial cell wall and is required for correct cellular morphology and opposition to osmotic forces. Despite extensive biochemical and genetic characterization of the enzymes responsible for the synthesis and degradation of PG (reviewed in [3], [4]), the mechanism by which these enzymes coordinate their activities remains poorly defined. It is clear, however, that dysregulation of this homeostatic balance frequently has lethal effects on the bacterium—inactivation of peptidoglycan synthases, either through the use of penicillin derivatives or overexpression of dominant negative forms of PG synthetic enzymes, induces lysis of cells [5], [6]. In many cases, this lethality can be suppressed by inactivation of several peptidoglycan hydrolases [5], [7], suggesting that PG hydrolase autolysin activity is restrained by functional interactions with PG synthases. This idea is consistent with a ‘make-then-break’ approach to cell wall synthesis where new PG subunits are first incorporated before the existing sacculus is cleaved to allow expansion [8]. One example of this is the formation of the septal PG—cells ensure that the septal PG is formed before PG hydrolases cleave apart the daughter cells.

Recent work suggests that the activity of PG synthetic and hydrolytic enzymes is regulated by the formation of protein complexes. In *E. coli*, the PG amidases AmiA, AmiB and AmiC can interact with non-enzymatic partners that upregulate septal peptidoglycan hydrolysis [9]. Conversely, the major bifunctional PG synthases, PBP1A and PBP1B in *E. coli* interact with and rely on essential lipoprotein partners for function [10]. In addition to interactions with non-enzymatic partners, several affinity chromatography and genetic studies have identified interactions between PG modulating enzymes themselves [11]. While the exact interactions may be species-specific, in general, PG synthases can associate with both other PG synthases and with PG hydrolases. Likewise, PG hydrolases can form predicted hydrolytic complexes with other autolysins [11]–[13]. These results suggest a general paradigm where PG modulating enzymes of both similar and opposing functions assemble as multi-protein complexes that spatially and temporally coordinate PG synthesis and hydrolysis during bacterial growth and division. An immediate challenge is to translate the many identified interactions into functional *in vivo* effects on the growth and division of bacteria.

Previously, we have studied regulation of the essential *M. tuberculosis* PG hydrolase, RipA (Rv1477). RipA belongs to the NLPC/p60 family, and has been characterized as a D,L D-glutamate-diaminopimelic acid (DAP) endopeptidase that cleaves within the pentapeptide bridges of the PG sacculus, thereby removing cell wall crosslinks [14]. The RipA homologue in Listeria (P60) and in *Mycobacterium marinum* (IipA) can be deleted, but this causes septal resolution defects [15], [16]. In contrast, RipA is essential in *M. tuberculosis* [17], and depletion of RipA produces a chaining phenotype in *M. smegmatis*, which causes severe growth inhibition [18]. This is unlike the case in *E. coli*, where extensive chaining and growth inhibition requires inactivation of several PG hydrolases [19].

In this work, we interrogate the mechanism by which RipA activity is regulated *in vivo* during vegetative growth. We report that RipA requires careful control to support growth and division without compromising the cell's structural integrity—RipA becomes a lethal autolysin when its activity is dysregulated. Under physiological conditions, RipA relies on protein interactions to correctly control its degradative capacity. These interactions are also necessary for proteolytic cleavage of RipA to produce active enzyme. RipA cleavage and activation is more robust in *M. smegmatis* than in the pathogenic *M. tuberculosis* or *M. bovis* BCG, which may be a reflection of the different PG hydrolysis requirements between fast and slow growing mycobacteria. However, bypassing RipA cleavage by overexpressing fully active truncated enzyme compromises the structural integrity of both *M. smegmatis* and *M. tuberculosis*, suggesting that RipA cleavage may be rate-limited in order to synchronize PG hydrolysis with the growth rate of the bacterium. These results suggest a model in which RipA is regulated by several interconnected post-transcriptional mechanisms—proteolytic processing produces active enzyme, while protein-protein interactions upstream and downstream of cleavage ensure RipA functions correctly at the septum.

## Results

### Dysregulated RipA functions as a lethal autolysin

**Figure 1:**
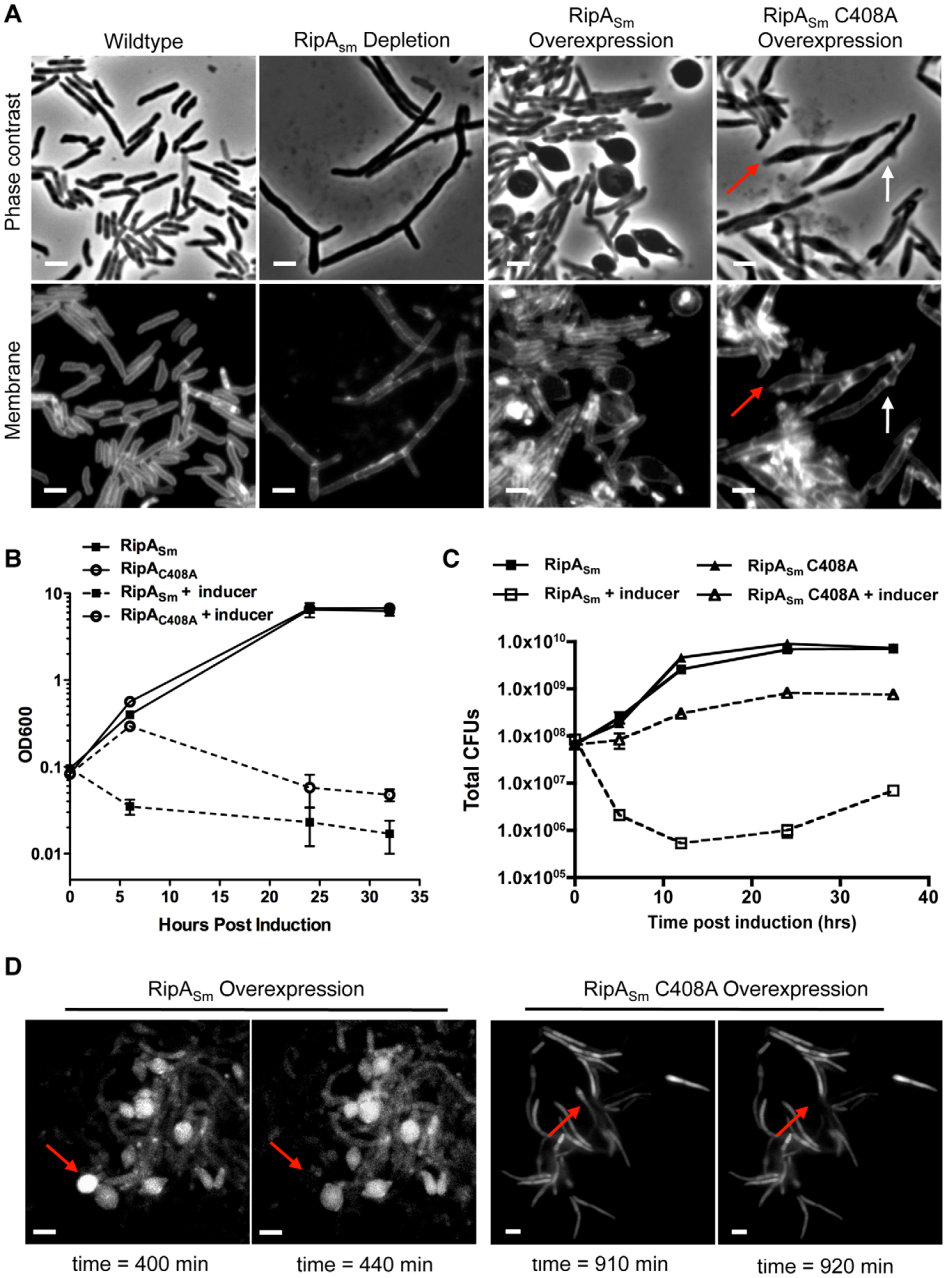
Dysregulation of RipA in *M. smegmatis* causes growth attenuation. (A) Micrographs of RipA dysregulated strains after 24 hours of depletion or induction. RipA depletion was achieved by placing *ripA* under the control of an anhydrotetracycline (aTc) inducible promoter [18]. Depletion occurred over 24 hours by growing this strain in the absence of aTc. Alternately, wildtype RipA (RipA_Sm_) or catalytically inactive (RipA_Sm_ C408A) *M. smegmatis* RipA was overexpressed in wildtype cells. In the C408A overexpression strain, both chaining (white arrows) and bulging (red arrows) cells were observed. Membranes were stained with TMA-DPH. Scale bar represents 2 µm. (B) *M. smegmatis* strains overexpressing wildtype (RipA_Sm_) and catalytically inactive (C408A) RipA were induced with aTc and growth assessed by OD_600_ over time. (C) *M. smegmatis* was induced with aTc to overexpress either wildtype RipA_Sm_ or inactive RipA_Sm_ C408A. Colony forming units (CFUs) were enumerated at the indicated time course by serial dilution and plating onto inducer-free agar. (D) Time-lapse microscopy was used to visualize *M. smegmatis* grown on an agar pad with aTc to overexpress wildtype RipA_Sm_ (left panels) or catalytically inactive RipA_Sm_ C408A (right panels). A GFP reporter was used to visualize RipA induction as well as determine cytokinesis and cell lysis. Presented here are frames from agar pads at four time points post-induction, showing individual cells before and after cell lysis, as detected by loss of GFP signal (arrows). Scale bar represents 2 µm.

When RipA is depleted, daughter cells are unable to separate and instead, grow as chains (Figure 1A). While cells require peptidoglycan hydrolysis to accomplish cell separation, excessive cell wall degradation can compromise structural integrity and lead to lysis. We hypothesized that RipA sits in this precarious situation, where the cell cannot tolerate either too little or too much RipA activity.

We investigated whether excessive RipA activity is toxic to mycobacteria by inducing *M. smegmatis* RipA (RipA_Sm_) from a tetracycline-inducible episomal plasmid in *M. smegmatis*. Unlike the chaining phenotype we have previously observed with RipA depletion, RipA overexpression caused the rod-shaped cells to become spherical and lyse, (Figure 1A, 1D (time-lapse movie in Video S1). This is dependent on catalytic activity, as overexpression of a catalytic mutant, RipA_Sm_ C408A, does not display this phenotype (Figure 1A, 1D, (time-lapse movie in Video S2). The spherical phenotype of RipA_Sm_ overexpression led to a severe growth defect by optical density (Figure 1B) and over one hundred fold killing, as determined by CFU enumeration (Figure 1C). Thus, excessive RipA activity in the cell is highly lethal.

### RipA depletion protects against PBP inhibition

To determine whether a more physiological level of RipA could be converted to a lethal autolysin, we dysregulated RipA activity through the use of the beta-lactam antibiotic meropenem. Beta-lactam antibiotics block PG precursor incorporation, which causes excessive PG hydrolase activity and cell lysis [7]. While *M. tuberculosis* is relatively resistant to most beta-lactams, recent work has shown that meropenem is more resistant to the endogenous mycobaterial beta-lactamase, and is highly effective at killing *M. tuberculosis*, especially in combination with the beta-lactamase inhibitor clavulanate [20]. Meropenem targets PBP2 and PBP3 in *E. coli*, as well as L,D transpeptidases in *M. tuberculosis* [21], [22]. Since RipA is known to interact with the PG synthase PBP1, which is required for normal vegetative growth [23] and morphology in mycobacteria [24] (depletion of the protein leads to rounded cells), we asked whether meropenem treatment can dysregulate RipA and convert the enzyme into a lethal autolysin.

**Figure 2:**
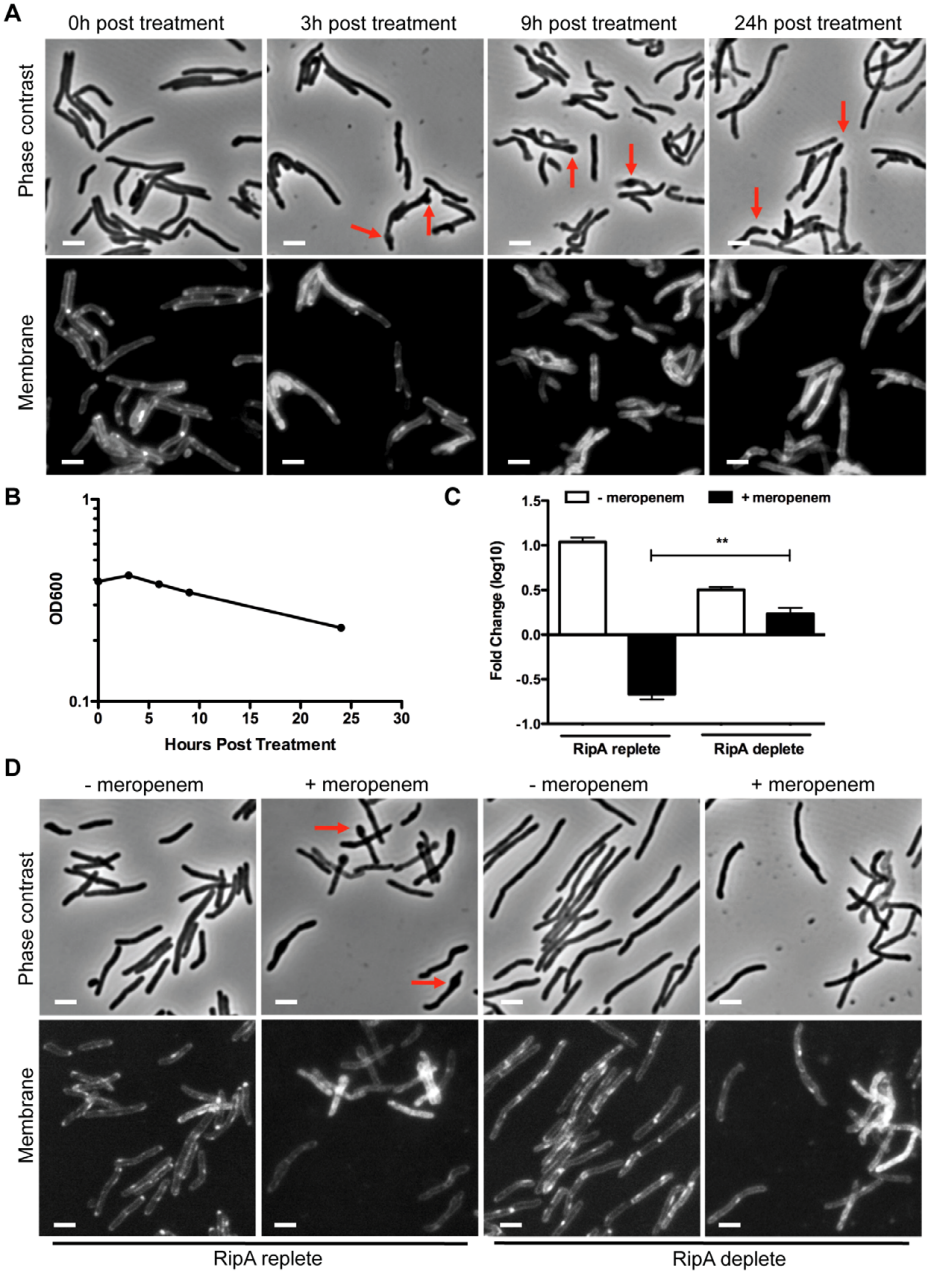
RipA depletion protects cells from meropenem-induced lysis. (A) A RipA conditional depletion strain of *M. smegmatis* was grown in the presence of inducer (RipA replete conditions) and 10 µg/mL of meropenem. Cells were imaged for morphological changes at various time points after meropenem addition. During meropenem treatment, septal and polar bulging were observed (arrows). Cell membranes were stained with FM4-64. Scale bar represents 2 µm. (B) Lysis of *M. smegmatis* cells from (A) was also characterized by OD_600_ at various times post meropenem treatment. (C) The RipA conditional depletion strain of *M. smegmatis* was grown with anhydrotetracylcine (aTc) inducer (RipA replete) or pre-depleted (RipA deplete) in the absence of aTc for 6 hours. These cells were then treated with 10 µg/mL of meropenem for 6 hours, washed, serially diluted and plated for CFU enumeration. (**, the difference between survival in RipA replete cells and RipA depleted cells in the presence of meropenem was significant with a p-value = 0.0006). (D) Cells post meropenem treatment in (C) were imaged by fluorescence microscopy to determine morphological changes. The plasma membrane was visualized with FM4-64. Scale bar represents 2 µm.

We first treated *M. smegmatis* with 10 µg/mL meropenem and assessed morphological changes over time by microscopy. Treated cells filament and swell at the poles and septa, which are the sites of mycobacterial PG incorporation (Figure 2A, arrows). This morphological toxicity correlated with a decrease in optical density over time, which suggested lysis (Figure 2B). This was borne out by CFU analysis, which showed that 80% of treated cells were killed within 6 hours of meropenem treatment (Figure 2C, bar 2).

The bulging at sites of PG incorporation after meropenem treatment suggested an excess of PG hydrolase activity. Since RipA localizes both to poles and septa, we hypothesized that RipA may play a role in killing meropenem treated cells. To test this idea, we depleted *M. smegmatis* of RipA before meropenem treatment (Figure S1A) and then assessed survival with meropenem treatment by CFU enumeration. We found that unlike RipA replete cells (Figure 2C, bar 2), meropenem did not kill RipA depleted cells (Figure 2C, bar 4). Furthermore, while RipA replete cells bulged under meropenem treatment as expected (Figure 2D, arrows), the RipA depleted cells appeared refractory to swelling (Figure 2D). As a control, cells were treated with SDS (which causes non-specific cell wall and membrane stress), as well as streptomycin, which targets protein synthesis. These control cells showed no survival (Figure S1B) or morphological (Figure S2) differences between RipA replete and depleted cells, demonstrating RipA specifically interacts with meropenem-affected pathways. Given that RipA enzymatic activity is modulated through protein-protein interactions with different PG synthetic and hydrolytic partners [18], [24], the meropenem data suggest that RipA is held in check in complexes by an interacting protein, such as PBP1. Furthermore, although there are many hydrolases that could contribute to cell death when PG synthesis is blocked, we found that at least for the synthases blocked by the clinically relevant beta-lactam antibiotic meropenem, RipA is quantitatively the single most important hydrolase.

### RipA post-transcriptional processes are required for septal resolution

Our results demonstrate that RipA dysregulation is highly detrimental to the cell. Thus, mycobacteria must control the activity of RipA during growth—there must be enough PG hydrolase activity around to support growth and division, but not an excessive amount so as to compromise structural integrity. One way this control may be regulated is at the transcriptional level.

We assessed whether the cell downregulates RipA expression using quantitative PCR. Since RipA is required for septation, which does not occur in non-replicative conditions, we compared RipA expression between exponential and stationary phases. We found that RipA remained expressed from exponential phase through the transition into stationary phase (Figure S3), suggesting there may be post-transcriptional mechanisms responsible for restraining RipA activity when it is not needed. Thus, we investigated whether achieving tight control of RipA activity may rely on post-transcriptional processes.

We hypothesized that removal of wildtype RipA from its endogenous niche *in vivo* would inhibit correct septal resolution and therefore phenocopy RipA depletion. If RipA requires downstream interactions for activity, e.g. with members of septal complexes or with post-translational enzymatic regulatory proteins, then we should be able to create a dominant negative RipA mutant, where the critical catalytic cysteine [25] is mutated to a nonfunctional alanine. Overexpression of the RipA_Sm_ C408A catalytic mutant should result in competition between nonfunctional RipA and endogenous RipA for required post-translational activation processes. If RipA requires these interactions to function, then we would observe chaining. Indeed, when we induced RipA_Sm_ C408A, cells grew as short chains (Figure 1A, white arrows), suggesting that RipA interactions are necessary for correct septal PG hydrolysis.

While overexpression of the RipA_Sm_ C408A mutant produced a severe growth defect (Figure 1B, 1C), it was not accompanied by the widespread lysis that was observed upon wildtype RipA_Sm_ overexpression (Figure 1C). The apparent drop in optical density upon longer induction of the RipA_Sm_ C408A strain (Figure 1B) was due to clumping of the culture, which though affecting optical density, did not lead to a drop in CFU, indicating growth inhibition rather than lysis (Figure 1C). However, we did observe occasional lysis of the RipA_Sm_ C408A strain in addition to the dominant negative chaining phenotype. Some cells within a chain produced a slight bulging phenotype, which is indicative of an increase in PG hydrolytic activity (Figure 1A, red arrows). These bulging cells, like in RipA dysregulated cells, can go on to lyse (Figure 1D, Video S2), though this does not lead to detectable cell death by CFU enumeration (Figure 1C). It is possible that displacement of endogenous wildtype RipA from complexes at the septum leads to activity at ectopic sites in a subset of cells. Alternatively, RipA overexpression could stimulate other endogenous PG hydrolases.

### RipA is proteolytically processed *in vivo*

**Figure 3:**
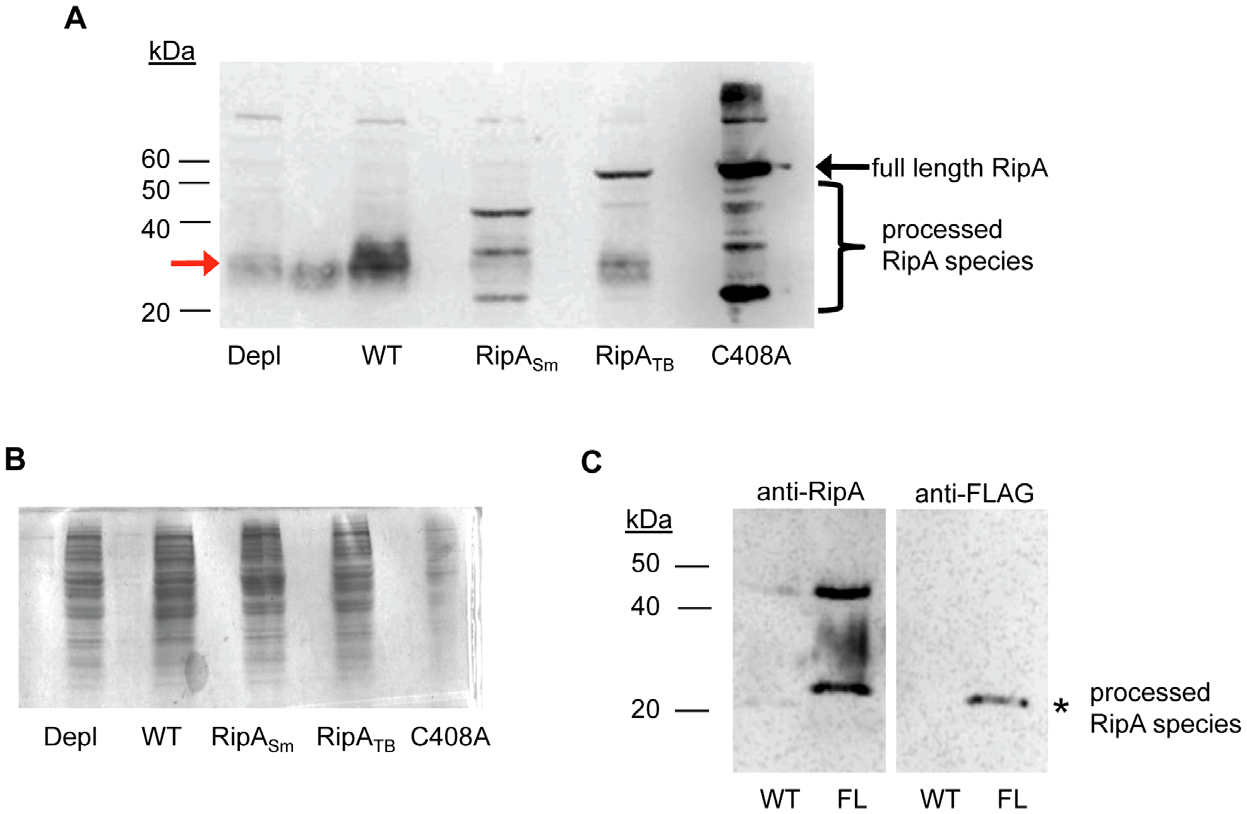
RipA_Sm_ but not RipA_TB_ is proteolytically processed in *M. smegmatis*. (A) Whole cell lysates were made from cells depleted for RipA (Depl), wildtype *M. smegmatis* (WT), and cells overexpressing either wildtype RipA_Sm_ (RipA_Sm_), RipA_TB_ (RipA_TB_), or catalytically inactive RipA_Sm_ C408A (C408A). The lysates were probed with anti-RipA polyclonal antibody. Full length RipA (arrow), and proteolytically cleaved endogenous RipA (red arrow) and recombinant species (brackets) were detected. (B) Coomassie stain of RipA-expressing *M. smegmatis* cells showing total protein loaded. Lanes are identical to (A). As RipA_Sm_ C408A induction is particularly strong, this sample was deliberately underloaded to prevent its signal in (A) from overwhelming detection of less abundant species. (C) Western blots with anti-RipA (left panel) and anti-FLAG antibodies (right panel). Culture filtrates were collected from wildtype *M. smegmatis* (WT) and cells in which chromosomal RipA was C-terminally fused to a FLAG epitope (FL). A truncated RipA species that is shifted by the FLAG tag was observed (asterisk).

When we used a RipA polyclonal antibody that recognizes a C-terminal epitope, we observed truncated RipA species from mycobacteria by Western blotting. When we overexpressed RipA_Sm_, we found several bands smaller than the predicted full length protein (Figure 3A, lane 3). Likewise, we saw these truncated bands when we overexpressed RipA_Sm_ C408A in *M. smegmatis* (Figure 3A, brackets). These products were not due to non-specific cytoplasmic degradation of overexpressed RipA, as we fractionated RipA_Sm_ C408A overexpressing cells and found that RipA processed species were enriched in the cell wall fraction (Figure S4A). The efficiency of fractionating mycobacteria was confirmed by Western blotting against RpoB (cytosolic) and mycobacterial antigen 85 (cell wall) markers (Figures S4B, S4C). Thus, these results suggest that RipA undergoes physiological post-translational processing in the periplasmic or cell wall compartment.

To further demonstrate that RipA processing is physiological and not an artifact of overexpression, we used Western blotting to estimate the size of RipA in wildtype *M. smegmatis* whole cell lysates. In mid-exponential phase cells, RipA formed a smear of ∼30 kDa (Figure 3A, lane 2, red arrow) with no detectable full length protein present. This signal was specific for RipA, as cells depleted for RipA (Figure 3A, lane 1) had decreased signal compared to wildtype cells (Figure 3A, lane 2) when equal amounts of total protein were analyzed (Figure 3B). Furthermore, processed endogenous RipA partitioned to the cell wall compartment (Figure S4A). The smear of processed RipA suggests there are multiple processing sites. This may represent multiple cleavage products or further modifications to the protein. A recent crystal structure of RipA suggests a protease labile loop exists between the N inhibitory and C terminal PG hydrolase domains; this loop is hypothesized to be the site of cleavage *in vitro*, which is required for RipA enzymatic activation [25]. We mutated candidate residues in the loop in an attempt to identify cleavage sites but were unsuccessful in blocking RipA processing (Figure S5).

Truncated RipA species were found associated with the cell wall compartment and in culture filtrates. In the culture filtrate, a RipA fragment appeared as a single band at approximately 25 kDa (Figure 3C, asterisk). We demonstrated this signal was specific by C-terminally tagging endogenous RipA on the chromosome of *M. smegmatis* with a FLAG epitope. This 25 kDa species exhibited altered mobility due to the epitope and could be detected by both anti-RipA (Figure 3C, left panel) and anti-FLAG antibodies (Figure 3C, right panels). These results indicate that RipA exists physiologically in a smaller form than the predicted full length protein.

### RipA requires processing for activity

**Figure 4:**
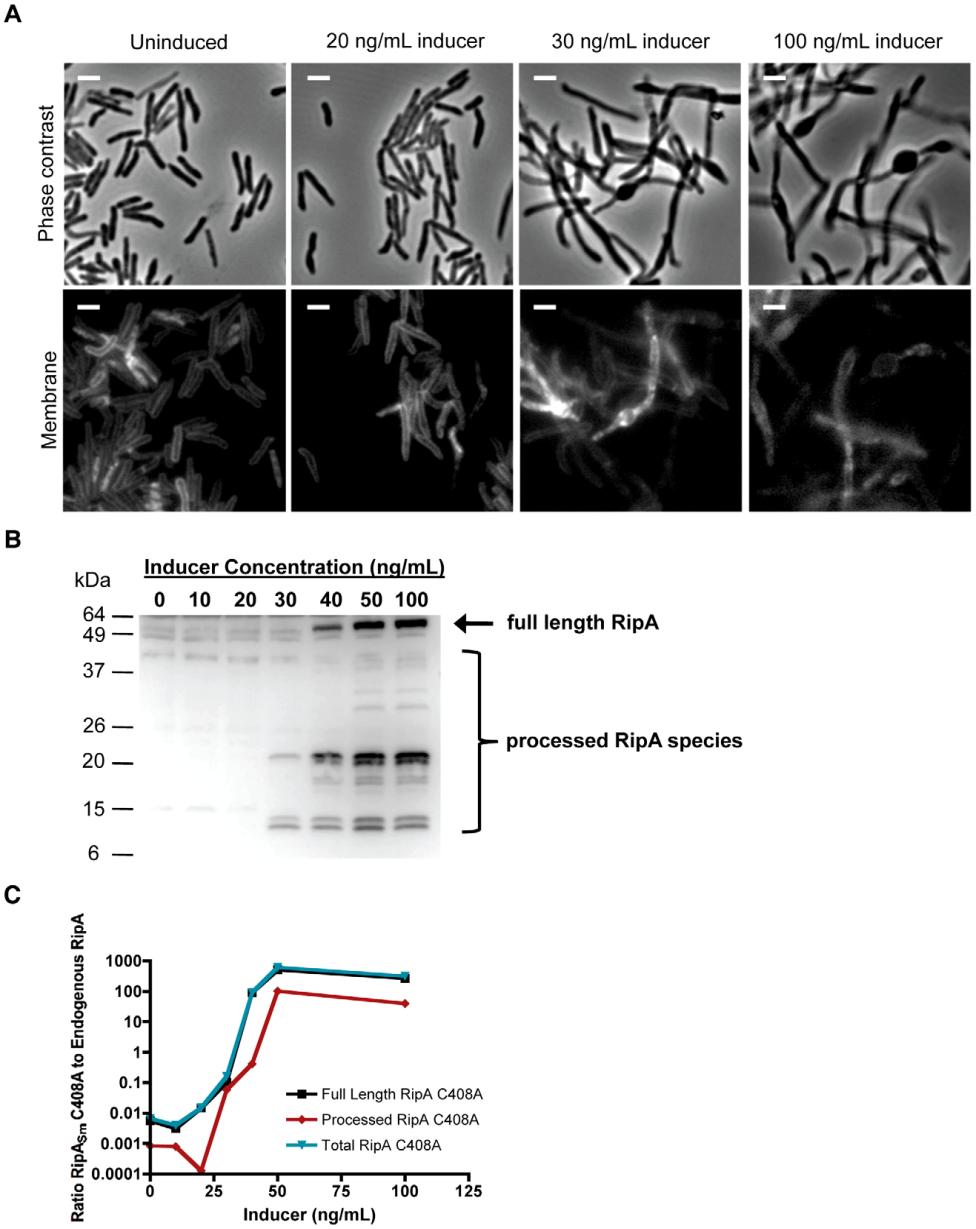
Proteolyzied RipA_Sm_ C408A overexpression correlates with morphological toxicity. (A) Micrographs of *M. smegmatis* grown in various concentrations of inducer to overexpress RipA_Sm_ C408A-FLAG. Scale bar represents 2 µm. (B) Total cell lysates from *M. smegmatis* overexpressing RIpA_Sm_ C408A-FLAG under various inducer concentrations were probed with anti-FLAG antibody. Both full length (arrow) and processed RipA_Sm_ C408A species (brackets) were detected, including two major processed species: a band at 25 kDa and a doublet around 12 kDa. (C) Samples from (B) were probed with anti-RipA antibody (Figure S7) and densitometry analysis performed. The levels of full length (black), processed (red) and total (blue) recombinant RipA_Sm_ C408A proteins were compared to endogenous RipA levels. The fold overexpression of RipA_Sm_ C408A relative to endogenous RipA was graphed as a function of inducer concentration.

The observation of RipA cleavage suggested this process could be required for the protein's function *in vivo*. To test whether RipA processing is correlated with division, we titrated overexpression of RipA_Sm_ C408A and quantified the amount of induction needed to mediate chaining. We found that low level overexpression with 30 ng/mL inducer was sufficient to cause chaining (Figure 4A) without saturating the processing machinery, since these cells did not accumulate full length RipA (Figure 4B). Instead, mildly overexpressed RipA was processed down to two sets of smaller species at around 23 kDa and 12 kDa (Figure 4B). We also saw a dose-dependent saturation of the endogenous processing capacity, with high induction leading to accumulation of full length RipA (Figure 4B), as well as loss of processed endogenous RipA (Figure S6A). When we quantified recombinant protein levels by comparing densitometry with endogenous RipA protein (Figure 4C), we found that even mild RipA_Sm_ C408A overexpression at 30 ng/mL of inducer (processed recombinant protein is approximately 10% of endogenous wild type RipA levels) was sufficient to cause chaining and cellular toxicity (Figure 4A). Together with qPCR data showing that RipA_Sm_ C408A induction does not affect endogenous RipA transcription (Figure S6B), these data suggest direct competition between the RipA_sm_ C408A mutant and endogenous RipA for processing machinery, and that the processed, not full length, form of RipA is required for division.

**Figure 5:**
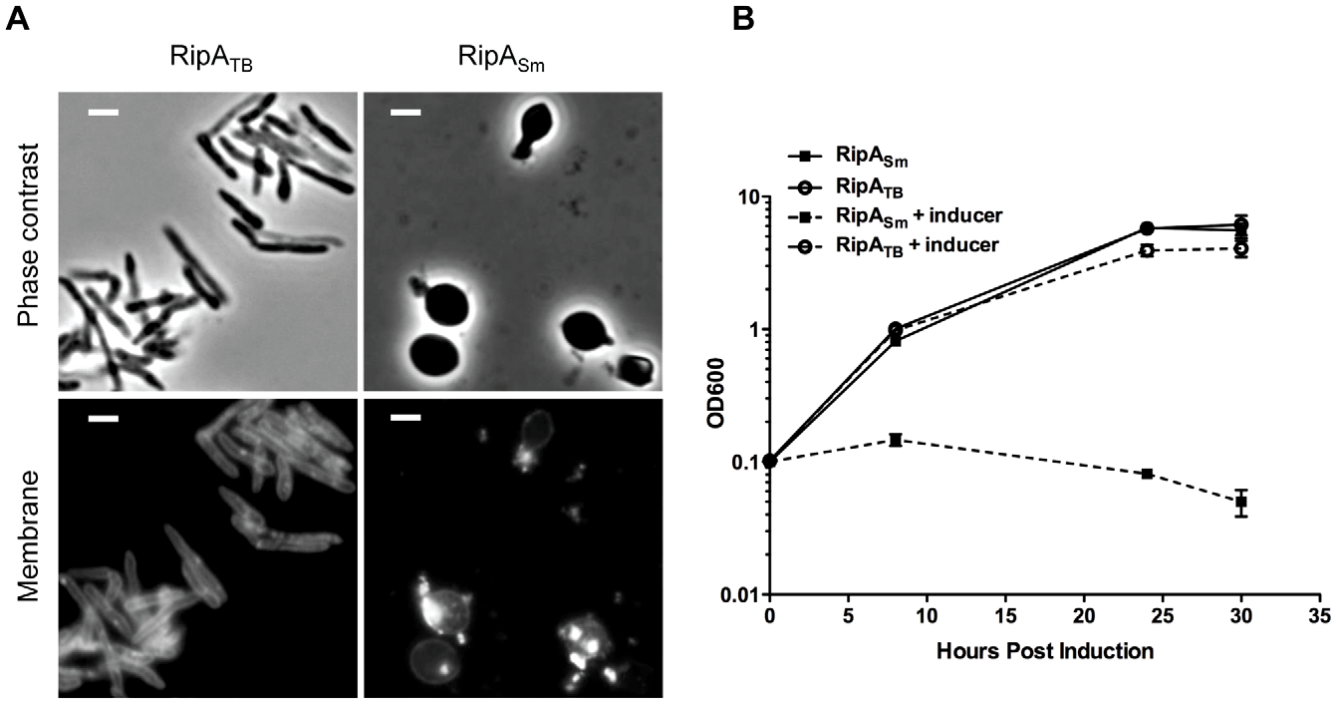
RipA is differentially active in a species-specific manner. (A) Fluorescence micrographs of *M. smegmatis* strains induced with aTc to overexpress *M. tuberculosis* RipA (RipA_TB_) or *M. smegmatis* RipA (RipA_Sm_). Membranes were stained with TMA-DPH. Scale bar represents 2 µm. (B) *M. smegmatis* strains in (A) were induced with aTc to overexpress RipA_TB_ and RipA_Sm_ constructs. Growth of these strains was monitored over time by OD_600_.

However, though correlated with function, the processed species we observed could be the product of an inactivating event. To test this, we took advantage of the observation that the *M. tuberculosis* homologue of RipA (RipA_TB_) functions differently in *M. smegmatis* than its native counterpart, despite having the same general domain architecture (Figure S7). In contrast to RipA_Sm_, which is toxic when overexpressed, overexpression of RipA_TB_ in *M. smegmatis*, surprisingly caused no toxicity or cell morphological differences (Figure 5A,B), despite similar protein levels (Figure 3A). We examined whether RipA_Sm_ toxicity was correlated with its processing by performing Western blot analysis on RipA_TB_ overexpressing *M. smegmatis*. In contrast to overexpression of RipA_Sm_, when wildtype RipA_TB_ is overexpressed we observed only a single full length band at 55 kDa (Figure 3A, right arrow). The absence of processing correlates with the lack of detectable RipA_TB_ toxicity in *M. smegmatis*. Thus, we hypothesize that proteolytic cleavage is required for activating RipA *in vivo*.

**Figure 6:**
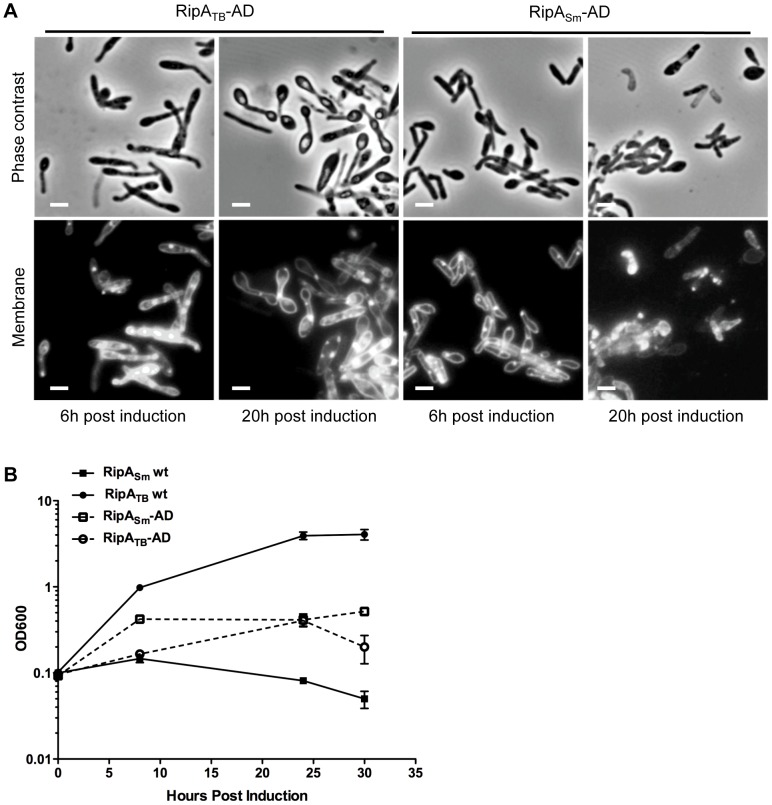
Truncation causes RipA_TB_ to become active in *M. smegmatis*. (A) *M. tuberculosis* and *M. smegmatis* RipA were truncated to the active endopeptidase domain (AD) and fused to the RipA secretion signal to generate RipA_TB_-AD and RipA_Sm_-AD. Micrographs of *M. smegmatis* overexpressing RipA_TB_-AD or RipA_Sm_-AD were taken at 6 and 20 hours post induction with aTc. Membranes were visualized by FM4-64 staining. Scale bar represents 2 µm. (B) *M. smegmatis* strains were constructed to overexpress full length RipA_TB_ and RipA_Sm_ and truncated RipA_TB_-AD, RipA_Sm_-AD constructs under the control of aTc. Growth of these strains in the presence of inducer was assessed over time by OD_600_. These strains grown in the absence of inducer as controls are shown in Figure S8.

However, it could be formally possible that RipA_TB_ cannot recognize *M. smegmatis* peptidoglycan or is not intrinsically active enough in *M. smegmatis* to cause morphological defects. To test if RipA_TB_ can be enzymatically functional in *M. smegmatis*, we deleted the predicted N-terminal inhibitory segment by fusing the truncated active domain of RipA_TB_ to the RipA secretion signal peptide (RipA_TB_-AD). As a control, we also produced a construct in which the *M. smegmatis* RipA active domain (RipA_Sm_-AD) can be secreted. None of the strains produced growth defects when uninduced (Figure S8). As expected, RipA_Sm_-AD like full length RipA_Sm_, was fully functional when induced and disrupted cell wall integrity, leading to bulging of the cells and a concomitant growth defect (Figure 6A,B). When RipA_TB_-AD was secreted, we found that it was also functional and behaved in the same way as RipA_Sm_-AD (Figure 6A,B). Thus, the catalytic domain of RipA_TB_ can be active in *M. smegmatis*, but full length RipA_TB_ is not toxic because it does not undergo efficient processing in *M. smegmatis*.

### RipA processing in *M. tuberculosis* requires additional steps

**Figure 7:**
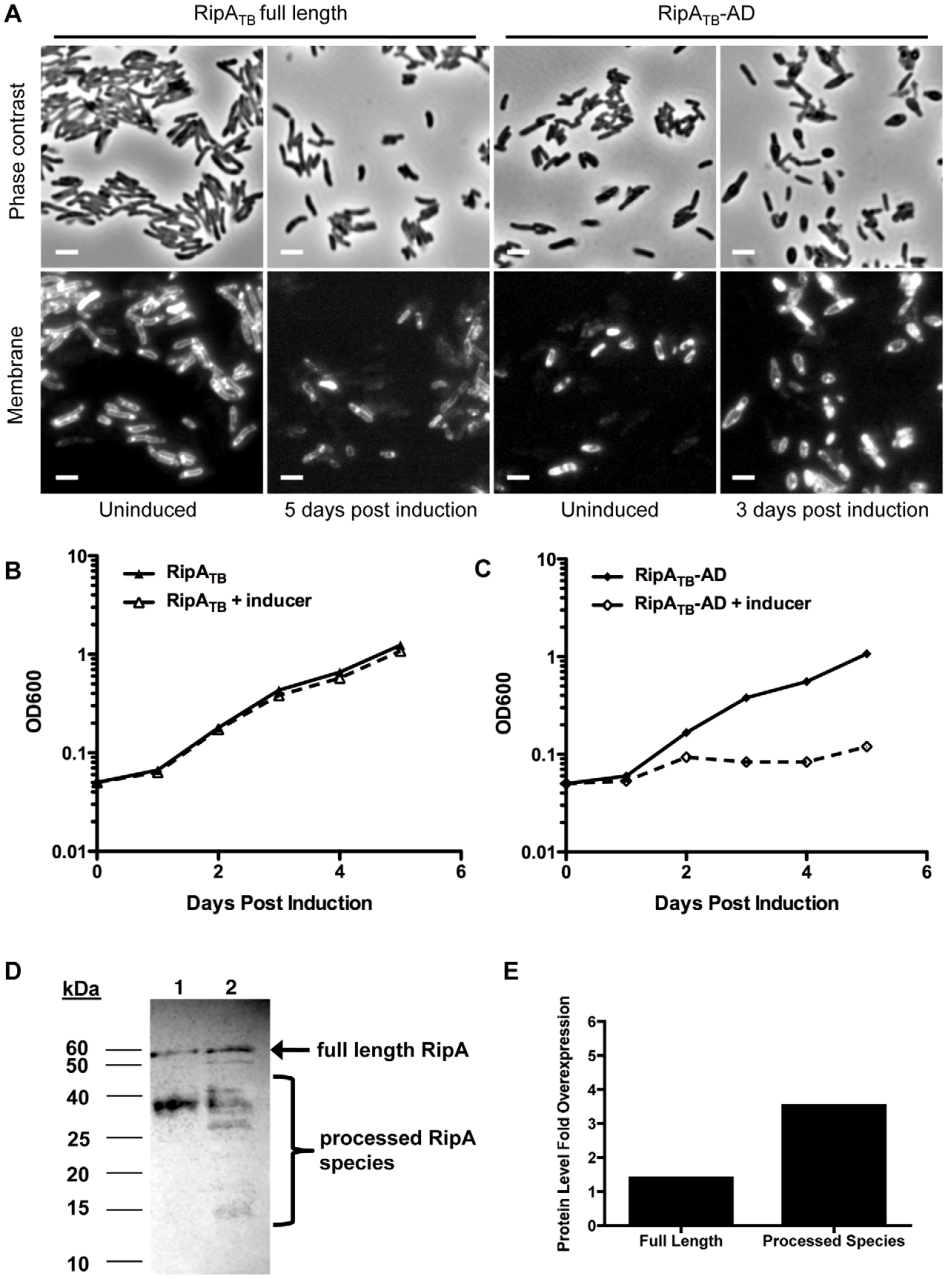
Processed RipA is toxic to *M. tuberculosis*. (A) Full length RipA_TB_ and truncated RipA_TB_-AD was overexpressed in *M. tuberculosis* for several days with addition of aTc. Cells were analyzed by microscopy for changes in morphology. Membranes were stained with FM4-64. Scale bar represents 2 µM. (B) Growth of *M. tuberculosis* induced with aTc and overexpressing full length RipA_TB_ was measured by OD_600_. (C) Growth of *M. tuberculosis* induced with aTc and overexpressing truncated RipA_TB_-AD was measured by OD_600_. (D) *M. tuberculosis* RipA (RipA_TB_) was overexpressed in *M. tuberculosis* by induction (lane 2) with aTc for 48 hours. RipA_TB_ was then detected by anti-RipA Western blot analysis. Uninduced lysates were used a control (lane 1). Full length RipA (arrow) and processed forms (brackets) were detected. (E) Total overexpressed protein was quantified from (D) by performing densitometry analysis on bands apparent in the induced strain that are absent from the uninduced control. Fold change in total RipA_TB_ overexpression relative to endogenous full length RipA signal was graphed.

Given the potentially toxic nature of hyperactive RipA we hypothesized that RipA processing and activation may be less robust in slow-growing mycobacteria in order to match their much slower rate of growth and consequent lower requirement for peptidoglycan hydrolysis. To investigate this model, we first determined whether RipA is processed in *M. tuberculosis* by overexpressing RipA_TB_. By Western blot analysis, we found multiple immunoreactive smaller species of RipA_TB_, suggesting processing in *M. tuberculosis* (Figure 7D, brackets). However, the induction of RipA_TB_ in *M. tuberculosis* did not produce morphological changes or growth defects, even after five days of induction (Figure 7A,B). This overexpression produced about 3 fold more protein (most of which is in the processed form) than endogenous full length RipA (Figure 7E), which is similar to the amount of overexpression needed to observe cell chaining in *M. smegmatis* with the RipA_Sm_ C408A allele (Figure 4C). The lack of morphological changes in *M. tuberculosis* is also in contrast with the marked lethality of RipA_Sm_ overexpression in *M. smegmatis* (Figure 1).

One explanation for this dichotomy may be that the slow growth of *M. tuberculosis* might mask morphological or growth defects caused by RipA overexpression. To test this possibility, we bypassed RipA processing by secreting truncated RipA_TB_-AD in *M. tuberculosis*. Induction of RipA_TB_-AD produced a severe growth defect in *M. tuberculosis* (Figure 7C), and concomitant cell rounding (Figure 7A) similar to that seen when RipA_Sm_ was overexpressed in *M. smegmatis*. These results show that unchecked RipA activity is toxic even to slow-growing mycobacteria. Since full length RipA_TB_ induction does not produce this toxicity, *M. tuberculosis* may have intrinsically less robust RipA processing than in *M. smegmatis*. Indeed, in *M. tuberculosis*, we observed a band of 55 kDa on Western blots probed with anti-RipA antisera. This band is the same size as full length RipA_TB_ and appears in both uninduced and induced samples (Figure 7D, arrow). As this form was not detected in wildtype *M. smegmatis* lysates (Figure 3A), it may represent endogenous, unprocessed full length RipA. The same full length band was also observed in *M. bovis* BCG cell lysates (Figure S9). These data, along with the active domain overexpression analysis, support the idea that slow-growing mycobacteria process RipA less efficiently in order to keep this potentially lethal activity in check.

## Discussion

Bacteria rely on peptidoglycan (PG) for shape and structure. The prevailing view of PG remodeling requires the concerted action of synthetic enzymes ligating new subunits into the existing PG lattice followed by hydrolysis of the PG sacculus by autolysins to allow cellular expansion or division. This process is accomplished through the action of large holoenzyme complexes in the periplasm consisting of both PG synthetic and hydrolytic enzymes. Disruption of PG synthesis in these complexes can dysregulate cognate PG hydrolases, which can then become autolysins that lyse the cell [26]. Thus, the coordination and regulation of PG hydrolases is a critical process for the survival of the bacterium.

Here we find that RipA in *M. tuberculosis* and *M. smegmatis* can behave as an autolysin, resulting in the formation of spherical cells and lysis when overexpressed or dysregulated. Overexpression of a RipA dominant negative mutant not only causes loss of septal resolution and chaining but also leads to uncontrolled activity of endogenous PG hydrolases and lysis in a subset of cells. Thus, RipA requires downstream interactions to govern its correct function during septal resolution, as well as prevent lethal ectopic hydrolase activity. The relatively low amount of dominant negative RipA (about 10% of endogenous RipA) required for chaining suggests that the cell has finely tuned the amount of active RipA in the cell to near the level required for division; even loss of 10% of these active RipA species (which is manifest in a partial loss of endogenous RipA processing (Figure S6A) leads to a block in septal resolution. While it is clear that RipA_Sm_ C408A overexpression can interfere with endogenous RipA activation, given its known interactions with two other PG remodeling enzymes that localize to the septum—RpfB and PBP1 [18], [24], [27]—it is possible the dominant negative mutant also incorporates into and inhibits functional PG remodeling complexes. A combination of these two activities may contribute to the RipA_Sm_ C408A mutant's potency at inducing chaining at relatively low levels of induction.

Supporting the presence of regulatory RipA septal complexes, we showed chemical inhibition of peptidoglycan incorporating PBPs (of which the RipA binding partner PBP1 is a member) results in cell rounding and lysis. Loss of PG synthetic activity within a PG remodeling complex may allow cognate PG hydrolases (such as RipA) to become hyperactive and lyse the cell. We found that RipA depleted cells were specifically protected against meropenem-induced killing, but remained sensitive to other unrelated stresses. The depletion of the RipA likely affects the expression of RipB, which resides downstream in the same operon and has the same *in vitro* enzymatic specificity as RipA [14]. However, RipB is not essential for growth [28], and we have previously shown that RipA appears to be more phenotypically active than RipB *in vivo*. RipA, but not RipB, can complement the growth inhibition and cell chaining defects observed in the *ripAB* depletion strain [18]. While we cannot discount the possibility that RipB contributes to meropenem-mediated killing, it seems more likely that RipA is the main enzyme responsible for this lethal phenotype. Together, our data suggests that meropenem-induced killing is RipA dependent. However, we do observe a slight but significant difference in growth between RipA depleted cells in the presence and absence of meropenem (Figure 2C, lanes 2 and 4) that suggests there may also be some RipA independent growth inhibition (but not lysis) due to meropenem treatment. This may reflect the fact that meropenem can target several transpeptidases [21], [22]. Despite this, since RipA appears to mediate meropenem's bactericidal capacity, and thus appears to be a more attractive target for drug development, we would expect that a chemical activator of RipA might act synergistically with meropenem treatment. From these data alone, it may be possible that a RipA inhibitor would be contraindicated in combination with meropenem, as it would antagonize the effect of PBP blockade, but we have previously observed that RipA depletion can sensitize cells to carbenicllin, a β-lactam antibiotic that also targets various transpeptidases [18]. In previous assays, in contrast to meropenem, carbenicillin sensitization required long term RipA depletion—it had no bactericidal effect on cells depleted for RipA in the same time scale as our meropenem studies (Figure S1B). These data suggest that extended treatment with a RipA inhibitor may weaken cells enough to cause sensitivity to PBP inhibitors to which the cell was previously resistant. It would be interesting to determine whether RipA blockade can, in fact, synergize with existing PG targeting antibiotics *in vivo*.

Because of the threat of lethal autolysin activity, cells can control PG hydrolases through several, interconnected regulatory mechanisms. RipA is no exception, as we have found that in addition to protein interactions that modulate its function, RipA requires proteolytic activation. RipA exists primarily as smaller processed forms in *M. smegmatis*. Recent work with RipA *in vitro* has mapped a protease labile loop between a putative N terminal blocking domain and the C terminal p60 PG hydrolase domain [25]. The size of our truncated RipA species could contain the predicted size of the p60 domain itself after cleavage within this loop, but we were unable to determine the exact cleavage site(s) for RipA proteolytic activation *in vivo* using site directed mutagenesis—mutation of two pairs of highly scissile aspartate-proline peptide bonds [29] at DP301 and DP315 to alanines had no effect on the ability of RipA to be cleaved in *M. smegmatis* (Figure S5). This is consistent with the activation of Auto amidase in *Listeria monocytogenes*, which is also produced as a zymogen and becomes active only after proteolytic processing and removal of an N terminal inhibitory domain [30]. For Auto it was not possible to isolate single amino acid substitutions that abolish processing; instead, only deletion of the loop prevented proteolytic cleavage [30], which suggests that the activation loop is intrinsically labile and might be cleaved by many different proteases. Like Auto amidase, RipA's labile loop can be cleaved by many proteases *in vitro* [25], and thus may be a target of several proteases *in vivo*. This may explain why we see a smear of RipA truncated species in wildtype mycobacteria, as opposed to a single truncated band.

Given the work of Ruggiero et al [25], it was likely that RipA is produced as a zymogen *in vivo*, like Auto amindase. However, another recent report suggested a different effect of the N terminal domain in blocking RipA enzymatic activity [14], [25]. While both studies agreed that the N terminal domain appears to partially block the C terminal endopeptidase active site, the authors reached opposite conclusions as to whether the N terminus is inhibitory. Ruggiero et al [25] found that truncated RipA containing only the C terminal p60 domain was able to cleave purified PG, while full length RipA had minimal activity [25]. In contrast, Böth et al [14] showed that full length RipA was capable of degrading small synthetic PG fragments, and truncation of the N terminus produced no increase in enzymatic activity. However, in the latter work, the authors did not perform enzymatic digests using full length RipA on purified PG, as performed by Ruggiero et al. It is possible that the reported differences between these studies reflects the ability of small PG fragments to enter the RipA active site, despite partial occlusion by the N terminal domain, while access of larger substrates such as crosslinked and polymerized PG is blocked. Our results favor the zymogen model, as we have found that processing of the N terminal domain is required for full RipA enzymatic activatio*n in vivo*. Likewise, the lack of processing of RipA_TB_ in *M. smegmatis* likely accounts for the absence of its toxicity upon overexpression. While it is possible that full length RipA could serve some degradation function on smaller substrates *in vivo*, our results suggest that its main peptidoglycan remodeling activity requires removal of the N terminus, which contains a functional inhibitory domain.

Furthermore, using the less efficiently processed RipA_TB_ homologue, we showed that protein interactions are not only necessary for regulating functional septal complexes but also promote RipA proteolytic activation. Full length RipA_Sm_ is toxic when overexpressed in *M. smegmatis*, but full length RipA_TB_ does not produce the same phenotype. However, when we bypassed processing and expressed the truncated RipA_TB_ active domain in *M. smegmatis*, we observed a full gain of toxicity. These results suggest that the interactions between RipA_Sm_ and the cellular factors necessary for processing do not occur with the RipA_TB_ homologue. Since septation is a highly conserved process, these data may not necessarily indicate different RipA binding partners in slow and fast growing mycobacteria but rather that the *M. smegmatis* and *M. tuberculosis* binding partners have evolved together and may have higher affinities for one another.

**Figure 8:**
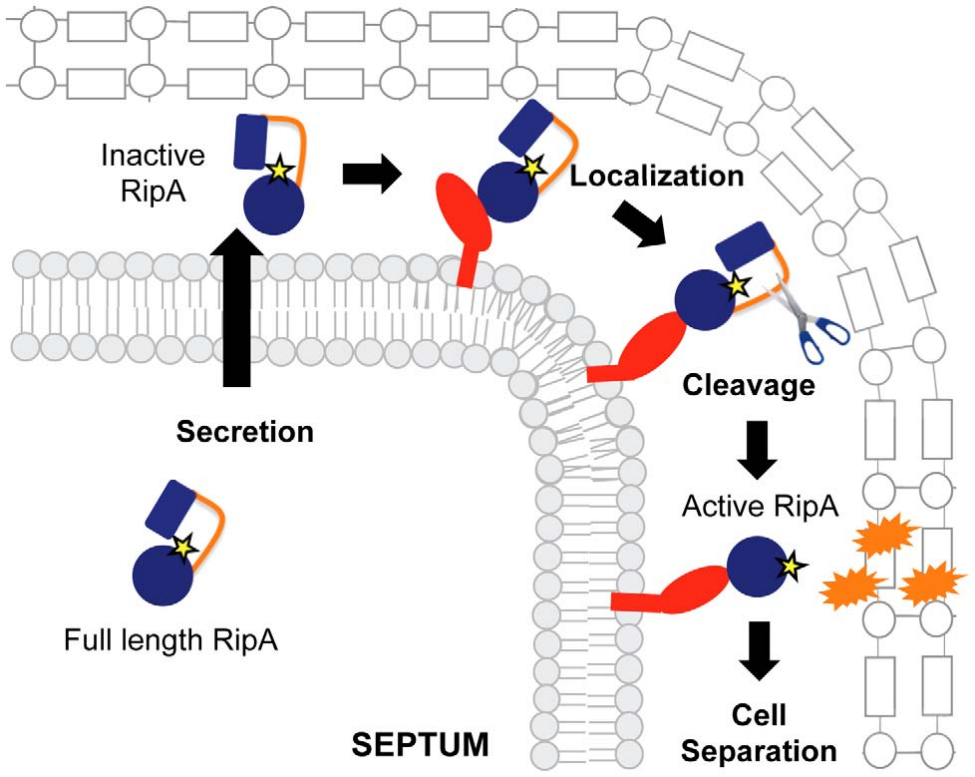
Model for RipA activation through proteolytic processing and protein interactions. RipA is produced as a full length zymogen with an N-terminal secretion signal. Once in the periplasm, RipA can bind to its interaction partners, leading to recruitment of proteases that cleave the extended loop between an N-terminal blocking domain and the C-terminal peptidoglycan hydrolase domain. The cleaved RipA C-terminal domain is now active and functions by cleaving the septal peptidoglycan, splitting the daughter cells, perhaps in conjunction with other hydrolases.

Together, our work demonstrates that RipA regulation occurs at multiple levels post-transcriptionally. We did not see any transcriptional downregulation in cells transitioning into non-replicating conditions (Figure S4). In fact, there was a significant increase in RipA transcription during the transition to stationary phase, but the functional consequence of this observation remains unknown. Furthermore, while overexpression of the dominant negative RipA_Sm_ C408A allele modulates processing of endogenous RipA (Fig S6A), this is due to competition for processing and not transcriptional feedback, even at high induction conditions (Figure S6B). This lack of transcriptional modulation is consistent with the observation that *ripA* expression has only limited variation across dozens of published experimental conditions (summarized on TBDB [31]), including general and antibiotic stresses. The only conditions under which *ripA* expression has been found to change are under non-replication conditions and, recently, when cells are blocked in cell division [32]. In the latter work, Plocinska *et al* found that *ripA* can be regulated by the MtrAB two-component system. Specifically, inhibiting septum formation prevents MtrB, which localizes at the septum, from activating the MtrA response regulator, leading to *ripA* downregulation. The authors proposed an interesting model in which *ripA* transcription could be upregulated by MtrB when it assembles at the division site; however, it remains unclear whether MtrAB regulation of *ripA* transcription occurs during normal growth or, instead, represents a stress response when cell division is inhibited. While the question of *ripA* transcriptional regulation during vegetative growth remains to be tested, our work suggests that post-translational mechanisms like processing may represent a key way of controlling RipA hydrolytic activity during growth. Therefore, we propose that protein-protein interactions help establish RipA function at the septum, where it is then aided in becoming proteolytically cleaved in the periplasm (Figure 8). After enzymatic activation, functional RipA can rely on both upstream and downstream protein interactions to help place it in the correct context during cell division—inhibition of cognate PG synthases can lead to dysregulated cell wall hydrolysis. The benefit of having multiple levels of RipA regulation is that the cell can exert a tighter control over RipA's activation and potential autolysin activity.

We found that RipA in *M. tuberculosis* is subject to less proteolytic activation than in *M. smegmatis*. The slow-growing mycobacteria like *M. tuberculosis* and *M. bovis* BCG might well have slower rates of PG hydrolysis and, consequently, reduced RipA activity. Indeed, we find significant amounts of full length RipA_TB_ in slow-growing mycobacterial lysates, a form that is not present in *M. smegmatis* lysates. Furthermore, while expression of the active domain of RipA_TB_ leads to severe growth inhibition with concomitant bulging, overexpression of full length RipA_TB_ has no such effect in *M. tuberculosis*, suggesting that slow-growing mycobacteria proteolytically activate less RipA than their fast-growing counterparts. The mechanism behind this additional control over RipA activation is not known, but may be at the level of expression or functional kinetics of the protease(s) responsible for RipA cleavage. In fact, there is an additional stretch of amino acids in RipA_Sm_ compared to RipA_TB_, which sits at the beginning of the N terminal inhibitory domain (Figure S7). Ultimately, an integrated mechanism for controlling PG hydrolases may represent a broad paradigm among cell wall degrading proteins. Multiple levels of regulation might be required to synchronize their activity to the cellular requirement while avoiding overactivity and toxicity. In support of this, expression of a dominant negative RipA allele at 10% of the endogenous RipA levels leads to abnormal chaining. Thus, *M. smegmatis* appears to carefully titrate the amount of processed RipA to nearly the minimum levels it requires for division.

Finally, beyond division mechanics, post-translational PG hydrolase regulation has the added benefit of inducing changes quickly in response to changing environmental conditions, especially in times of low transcription such as non-replicative conditions [33], [34]. The byproducts of PG hydrolysis can act as sensors for the bacterial environment, whether *in vitro* or within a host. For example, in *B. subtilis*, muropeptides have been found to be sufficient to induce spore resuscitation [35], [36] while in *M. tuberculosis*, RpfB, a lysozyme that is known to be a RipA interacting partner [27], is required for regrowth from both *in vitro* and *in vivo* non-replication states [37], [38]. The exact mechanism behind mycobacterial resuscitation remains unclear, but muropeptide-based signaling could play a major role. In fact, we found processed RipA species in the culture filtrates and recent work by Mir et al [39] demonstrated that the addition of muropeptides to dormant *M. tuberculosis* facilitated resuscitation, possibly through the binding and signaling of the essential mycobacterial integral membrane kinase, PknB. Thus, soluble PG remodeling proteins might play a role in fostering communication across a bacterial population.

In summary, this work has further defined two connected, but distinct, mechanisms to regulate the activity of RipA, a potential autolysin that is essential for septal resolution in mycobacteria. The complexity of this regulation, which involves protein interactions as well as proteolytic activation, underscores the importance of carefully coordinating cell wall hydrolysis during growth and division. By dissecting the molecular regulation of PG hydrolases, we gain fundamental insight into how the bacterial cell wall is dynamically maintained and also open up avenues for novel chemotherapeutics, especially against major human pathogens such as *M. tuberculosis*.

## Materials and Methods

### Strains and culture conditions

*E. coli* XL-1 Blue (Stratagene, Santa Clara, CA) were grown at 37°C in LB broth or agar and used for cloning. Selection was performed using kanamycin (50 µg/mL), hygromycin (100 µg/mL), ampcillin (100 µg/mL) or zeocin (25 µg/mL) when appropriate. *Mycobacterium smegmatis* mc^2^155 was grown at 37°C, unless otherwise indicated, in Middlebrook 7H9 broth supplemented with ADC (bovine albumin fraction V (Sigma)(5 g/L)-dextrose (2 g/L)-catalase (3 mg/L) and 0.05% Tween80. Selection of *M. smegmatis* was achieved by supplementation of kanamycin (25 µg/mL), hygromycin (50 µg/mL) or zeocin (25 µg/mL). *M. tuberculosis* H37Rv and *M. bovis* BCG were grown in liquid Middlebrook 7H9 broth and plated on Middlebrook 7H10 agar supplemented with OADC (oleic acid-albumin-dextrose-catalase) (BD Biosciences, Franklin Lakes, NJ). *M. smegmatis* in which the RipA endogenous promoter has been replaced by a tetracycline inducible promoter was previously constructed and characterized in [18].

### Recombinant DNA constructs

Mtb RipA (Rv1477) and Msmeg RipA (MSMEG_3145) mutants were constructed through PCR stitching using the following primers:

Mtb C383A RipA Forward (CCGTCGGCTTCGACGCCTCAGGCCTGGTGTTG)

Mtb C383A Reverse (CAACACCAGGCCTGAGGCGTCGAAGCCGACGG)

Msmeg RipA C408A Forward (ACCGTCGGCTTCGACgcCTCGGGTCTGATG)

Msmeg C408A Reverse (CATCAGACCCGAGgcGTCGAAGCCGACGGT).

Msmeg RipA DP301AA DP315AA double point mutants were constructed by PCR stitching using the following primers:

Msmeg RipA DP315AA Forward (GCGATCCCGAGCGCGTTCGTCAGCGGTGcCgCCATCGCGATCATCAAC)

Msmeg RipA DP300AA Reverse (GAACGCGCTCGGGATCGCAGGCAGGGTCgCGgCCCACACGGCCCAGTT)

Secreted, RipA catalytic domain constructs were made using the *M. smegmatis* RipA secretion signal amino acids 1–51—Reverse primer: GAACCTgatatcGACGAGCGTGGCGAG. The RipA_TB_ active domain contained amino acids 332–472, while RipA_Sm_ active domain contained amino acids 357 to 494.

Tetracycline inducible strains were created by cloning RipA genes into the Tet On plasmid, pSE100. For time-lapse microscopy, green fluorescent protein (GFP) was cloned downstream of RipA_Sm_ C408A to create a transcriptional reporter. GFP was also cloned downstream in frame with RipA_Sm_ to create a translational fusion. These inducible plasmids were then transformed into *M. smegmatis* in which the plasmid pMC1s, which encodes the *tetR* gene, had already been integrated at the L5 site.

### Protein expression

Recombinant gene products were expressed using a published anhydrotetracycline inducible system [40]. Anhydrotetracycline induction was performed with 100 ng/mL of anhydrotetracycline unless otherwise indicated.

### Meropenem treatment and CFU enumeration

*M. smegmatis* with chromosomal *ripA* under the control of a tetracycline inducible promoter was grown in 7H9 ADC in the presence of 100 ng/mL anhydrotetracycline (aTc) to an OD of 0.2. The culture was split, the cells pelleted at 5000 rpm for 10 minutes and resuspended in 7H9 ADC with (RipA replete) or without (RipA depleted) aTc for 6 hours. Once depletion in the no inducer culture was confirmed by microscopic examination of the culture for chaining, 10 µg/mL meropenem were added and the cultures and cells grown at 37°C for 6 hours. After 6 hours of meropenem treatment, both RipA replete and depleted cultures were serially diluted and plated onto LB supplemented with hygromycin, kanamycin and 100 ng/mL aTc. Plates were incubated for 3 days at 37°C and colonies were counted. As a control, RipA replete and depleted cells were also treated with 0.08% SDS (w/vol), 0.08 µg/mL streptomycin (Sigma) or 500 µg/mL carbenicillin (Sigma).

### Protein fractionation and preparation

*M. smegmatis* cells were grown overnight in 7H9 supplemented with dextrose (2 g/L), but not albumin or catalase at 37°C until mid log phase. This media should be made fresh for every experiment. Cells were pelleted and culture supernatants were precipitated with 10% TCA (tricholoroacetic acid) overnight at 4°C. Precipitates were pelleted at 15,000×g for 15 minutes at 4°C and washed once with ice cold acetone. The acetone wash was decanted, and the pellet was air dried at room temperature. Precipitated protein was resuspended in reducing SDS loading buffer at 65°C, for 10 minutes.

*M. smegmatis* cells was fractionated by French press three times. Unbroken cells and insoluble material were pelleted at 1000×g for 10 minutes. The supernatant was collected and insoluble cell wall material pelleted at 27,000×g at 4°C for 40 minutes. The remaining supernatant was centrifuged at 100,000×g for 1 hour at 4°C to pellet the membrane fraction, while te supernatant contains the soluble cytosolic fraction.

### Immunoblotting and densitometry analyses

Rabbit polyclonal antibody was made from an affinity purified using a peptide derived from the Msmeg RipA epitope: NAGRKIPSSQMRRG (Genscript, Piscataway, NJ). Anti-RipA antibody was diluted to 1 mg/mL and used at a dilution of 1∶1000. Anti-FLAG antibody (Sigma, St. Louis, MO) was used according to manufacturer's instructions. Protein samples were mixed with 4x Laemmli SDS PAGE buffer (Boston BioProducts, Inc, Boston MA) and boiled for 5 minutes. *M. bovis* BCG and *M. tuberculosis* protein samples were boiled for 20 minutes. Proteins were separated on 12% Tris-glycine polyacrylamide gels, transferred to PVDF membrane (Pall Corp, Pensacola, FL), probed with anti-sera and developed with SuperSignal chemiluminescent reagent (Thermo, Pittsburg, PA). Densitometry on Western blot signal was performed using Multiguage software (Fujifilm).

### Microscopy and imaging

For TMA-DPH (Invitrogen, Carlsbad, CA) staining, bacteria were centrifuged and media removed. Cell pellets were resuspended in 50 mM TMA-DPH in PBS and incubated in the dark for 10 minutes. Cells were also stained in FM4-64Fx (Invitrogen) at a concentration of 5 µg/mL in PBS for 10 minutes, and then fixed and stored in 4% paraformaldehyde. Samples were imaged using a Nikon TE-200E microscope with a 100x (NA1.4) objective and captured with an Orca-II ER cooled CCD camera (Hamamatsu, Japan). Shutter and image acquisition were controlled using Metamorph Software (Molecular Devices). Final images were prepared using Adobe Photoshop 7.0.

### Time lapse microscopy

Four Gene Frames (Fisher Scientific) were stacked onto a glass slide and filled with Middlebrook 7H9 in low melting point agar, supplemented with 50 µg/mL of hygromycin and 100 ng/mL aTc. A glass coverslip was flattened atop the agar to create a smooth surface and then removed after the agar set. The agar pad was sliced into eighths and seven of the pieces were removed to provide an air reservoir. Onto the remaining pad, exponential phase *M. smegmatis* was pipetted and allowed to adsorb until the surface of the pad appeared dry. Finally, a glass coverslip was applied and the slide was imaged on the microscope in an environmental chamber warmed to 37°C (Applied Precision, Inc.).

Time-lapse images were acquired using a DeltaVision epifluorescence microscope with an automated stage enclosed with a 100x oil objective (Plan APO NA1.40). Cells were imaged every 10 minutes for up to 18 hours using brightfield and fluorescence illumination (461–489 nm; Applied Precision, Inc.) and images recorded with a CoolSnap HQ2 camera (Photometric). Focus was maintained using the software-based autofocus (Applied Precision, Inc).

### Real time PCR

*M. smegmatis* samples were collected at the indicated growth phases (log, OD_600_ = 0.5; early stationary, OD_600_ = 2; Stationary, 24 hours of OD_600_>7) and stored in RNA Protect Bacteria Reagent (Qiagen, Valencia, CA) at −80°C. The pellets were then mechanically disrupted by beadbeatting for three 1-minute cycles, and RNA isolated using the RNeasy Mini Kit (Qiagen), with one additional DNAse treatment (Qiagen) on the column before elution and a second DNAse digestion with Turbo DNase according to manufacturer's instructions (Ambion, Foster City, CA). Reverse transcription was carried out using the High Capacity cDNA Reverse Transcription kit (Applied Biosystem, Foster City, CA). Quantitative PCR reactions were set up in Power SyBr green PCR master mix (Applied Biosystems) and run and analyzed on a Step One Plus real time system (Applied Biosystems).

*ripA* expression was measured using the following intragenic primers: 5′ CAGATCGGTGTGCCCTACTC; 5′ GGCGAACATGTAGAGCATCAG; or against the 3′ UTR region: 5′ GCTCGAGGCCCCTTACAC; 5′ GGAGCGCAAAGTAATCCCATCAG

*ripA* expression was normalized to *sigA* levels, which utilized the following primers: 5′ AAGACACCGACCTGGAACTC; 5′AGCTTCTTCTTCCTCGTCCTC.

## Supporting Information

Figure S1 **RipA depleted cells are not more resistant to general stress.** (A) The *M. smegmatis* RipA conditional depletion strain was grown in the presence or absence of inducer for 6 hours, after which, cells were confirmed to be depleted for RipA by microscopic visualization of short chains in the absence of inducer (red arrows). Membranes were visualized by FM4-64. Scale bar represents 2 µm. (B) RipA replete or pre-depleted cells were treated for an additional 6 hours with either 0.8 µg/mL streptomycin (Sm), 0.08% SDS or 500 µg/mL carbenicillin (Cb). After treatment, cells were serially diluted and plated for CFU. * = the difference between RipA replete and depleted growth or survival is significant with p-value of < 0.1. ns = p-value is not significant (>0.1).
(TIF)

Figure S2 **RipA depleted cells are not morphologically resistant to general stress.** RipA replete and pre-depleted *M. smegmatis* were treated with various chemical stresses (0.8 µg/mL streptomycin, 0.08% SDS or 500 µg/mL carbenicillin for 6 hours, stained with FM4-64 and morphology assessed by fluorescent microscopy. Scale bar represents 2 µm.
(TIF)

Figure S3 **RipA is not post-transcriptionally downregulated.** Quantitative PCR was performed against the *ripA* transcript during different growth phases of wildtype *M. smegmatis*. Expression was normalized to *sigA* transcripts in each sample. p-values: ns, non significant; *, p < 0.05.
(TIF)

Figure S4 **RipA processing is within the cell wall compartment.** (A) Anti-RipA Western blot of fractionated wildtype *M. smegmatis* (lanes 2), cells depleted for RipA (lanes 1) or cells overexpressing inactive RipA_Sm_ C408A (lanes 3). (B) Anti-RpoB Western blot of fractionated wildtype (lanes 1), RipA depleted (lanes 2) or RipA_Sm_ C408A overexpressing (lanes 3) *M. smegmatis*. (C) Anti-Ag85 Western blot of fractionated wildtype (lanes 1), RipA depleted (lanes 2) or RipA_Sm_ C408A overexpressing (lanes 3) *M. smegmatis*. Each lane was standardized to total protein from the cell wall fraction. Due to the heavy presence of recombinant RipA in RipA_Sm_ C408A induced cells, upon protein normalization, there is an enrichment for RipA protein, which leads to an apparent decrease in other cell wall proteins, such as Ag85.
(TIF)

Figure S5 **DP301 and DP315 residues are not necessary for RipA cleavage.** Site-directed mutagenesis was used to create a RipA_Sm_ DP300AA DP315AA double substitution mutant in the RipA_Sm_ C408A background. This mutant (DP300AA DP315AA C408A) was fused to a FLAG tag and expression induced with aTc in *M. smegmatis*. Total cell lysate was run on SDS-PAGE and RipA processing was monitored by Western blot analysis using an anti-FLAG antibody. RipA processed species are indicated in brackets and RipA full length is indicated by an arrow.
(TIF)

Figure S6 **Overexpression of RipA_Sm_ C408A is mild relative to endogenous RipA levels.** (A) The *M. smegmatis* RipA_Sm_ C408A overexpression strain was grown under various inducer concentrations and total protein harvested for Western blot analysis with anti-RipA antibody (top panel) and anti-ClpP antibody (loading control, bottom panel). Full length recombinant (arrow) and processed RipA C408A was detected (bottom brackets), as well as endogenous RipA (upper brackets). (B) Total RNA was harvested from cells grown in various concentrations of inducer and quantitative RT-PCR performed against endogenous RipA (primers detect the *ripA* 3′ UTR region) and normalized to *sigA* levels. ‘ns’ = not significant (p-value>0.1).
(TIF)

Figure S7 **Alignments between RipA homologues from** ***M. tuberculosis*** **and** ***M. smegmatis*** **.** (A) Amino acid alignment between RipA-TB from *M. tuberculosis* and RipA-smeg from *M. smegmatis*. Conserved residues are shown in black or denoted with ‘ * ’ in the consensus row, while shared amino acids with similar properties are in gray or marked with a ‘ . ’ in the consensus line. (B) Schematic diagram of the predicted domain structure of RipA_TB_ and RipA_Sm_ from *M. tuberculosis* and *M. smegmatis*, respectively. Both proteins have an N terminal secretion signal (black), a DUF or domain of unknown function (blue), an inhibitory domain (red), an extended loop (black line) and an NLPC/p60 family peptidoglycan hydrolysis domain (orange). RipA_Sm_ also has an additional 33 amino acid extension at the junction between the DUF and inhibitory domains (green box).
(TIF)

Figure S8 **Uninduced strains of** ***M. smegmatis*** **were equally viable.** *M. smegmatis* strains were constructed to overexpress full length RipA_TB_ and RipA_Sm_ and truncated RipA_TB_-AD, RipA_Sm_-AD constructs under the control of aTc. As negative controls, growth of these strains in the absence of inducer was assessed over time by OD_600_.
(TIF)

Figure S9 **RipA processing is rate limiting in slow growing mycobacteria.** (A) Full length RipA_Sm_ was overexpressed in *M. bovis* BCG for 48 hours. Cells were analyzed for changes in morphology by microscopy. Membranes were stained with FM4-64. Scale bar represents 2 µm. (B) Anti-RipA Western blot of *M. bovis* BCG induced to overexpress RipA_Sm_ (lane 2). Uninduced *M. bovis* BCG lysate was run as a control (lane 1). Full length RipA (arrow), as well as processed forms (brackets), were detected.
(TIF)

Video S1 **RipA_Sm_ overexpressed cells lyse.** *M. smegmatis* carrying a plasmid in which wildtype RipA_Sm_ was fused to GFP was grown on an agar pad with the inducer aTc, in order to overexpress wild-type RipA. Micrographs of the GFP signal were captured every 10 minutes by fluorescence microscopy and a time-lapse movie was compiled from the individual timepoints. Lysis of RipA expressing cells can be identified by loss of GFP between time points.
(AVI)

Video S2 **RipA_Sm_ C408A cells predominantly chain but also occasionally lyse.** *M. smegmatis* were grown on an agar pad, containing the inducer aTc in order to overexpress the catalytic mutant of RipA_Sm_ (C408A). A cytosolic GFP reporter was used to identify cells inducing RipA_Sm_ C408A expression and visualize chains of cells. Images were captured every 10 minutes, and a time-lapse movie was generated. Lysis of specific cells within the chain can be observed by localized loss of GFP signal between frames.
(AVI)
