# Supplementary figures and images for: Protein Complexes and Proteolytic Activation of the Cell Wall Hydrolase RipA Regulate Septal Resolution in Mycobacteria
Source: PLoS Pathog. 2013 Feb 28;9(2):e1003197. doi: 10.1371/journal.ppat.1003197 (PMC3585148; doi:10.1371/journal.ppat.1003197)

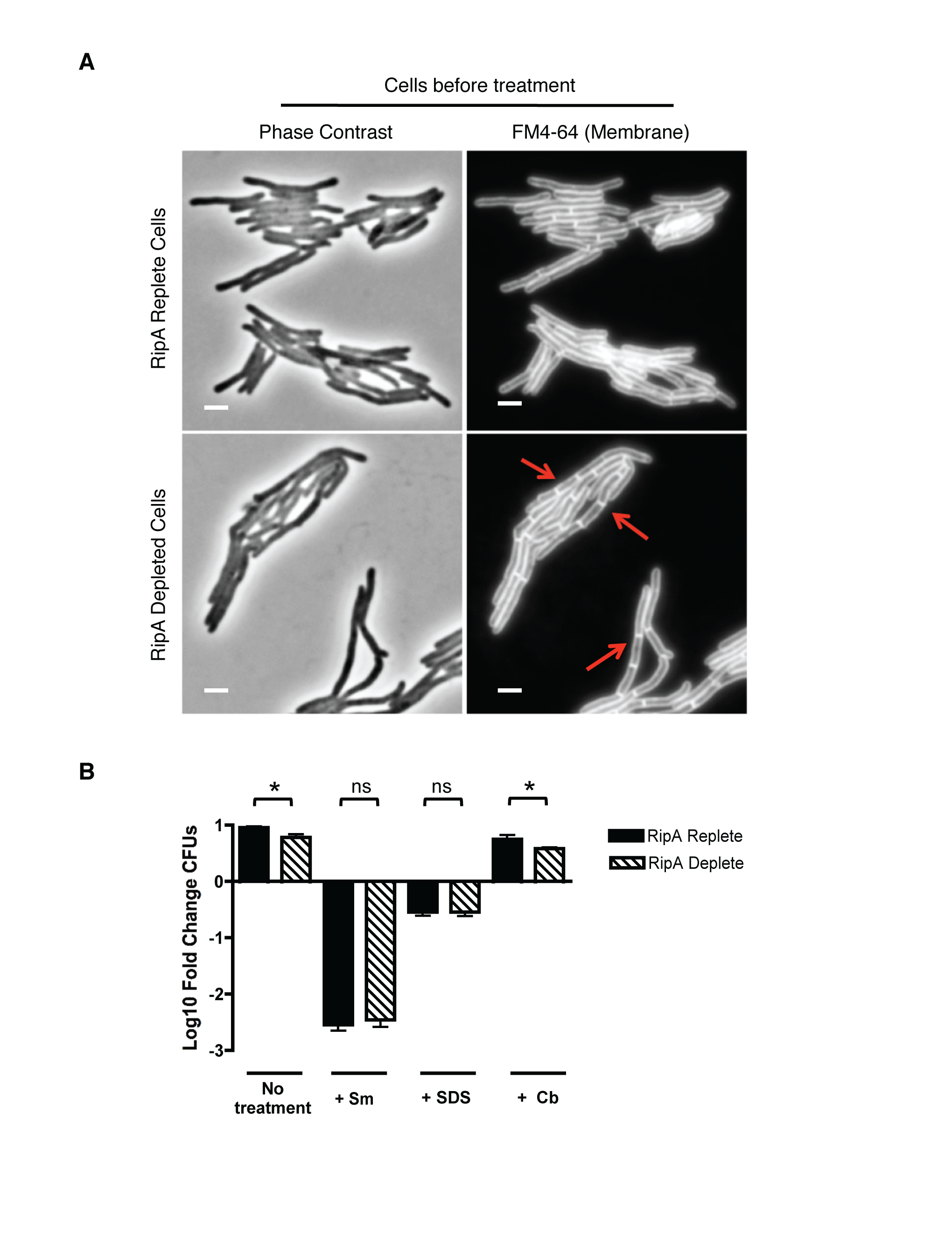

Supplement: Figure S1 — RipA depleted cells are not more resistant to general stress. (A) The M. smegmatis RipA conditional depletion strain was grown in the presence or absence of inducer for 6 hours, after which, cells were confirmed to be depleted for RipA by microscopic visualization of short chains in the absence of inducer (red arrows). Membranes were visualized by FM4-64. Scale bar represents 2 µm. (B) RipA replete or pre-depleted cells were treated for an additional 6 hours with either 0.8 µg/mL streptomycin (Sm), 0.08% SDS or 500 µg/mL carbenicillin (Cb). After treatment, cells were serially diluted and plated for CFU. * = the difference between RipA replete and depleted growth or survival is significant with p-value of < 0.1. ns = p-value is not significant (>0.1). (TIF) [file ppat.1003197.s001.tif]

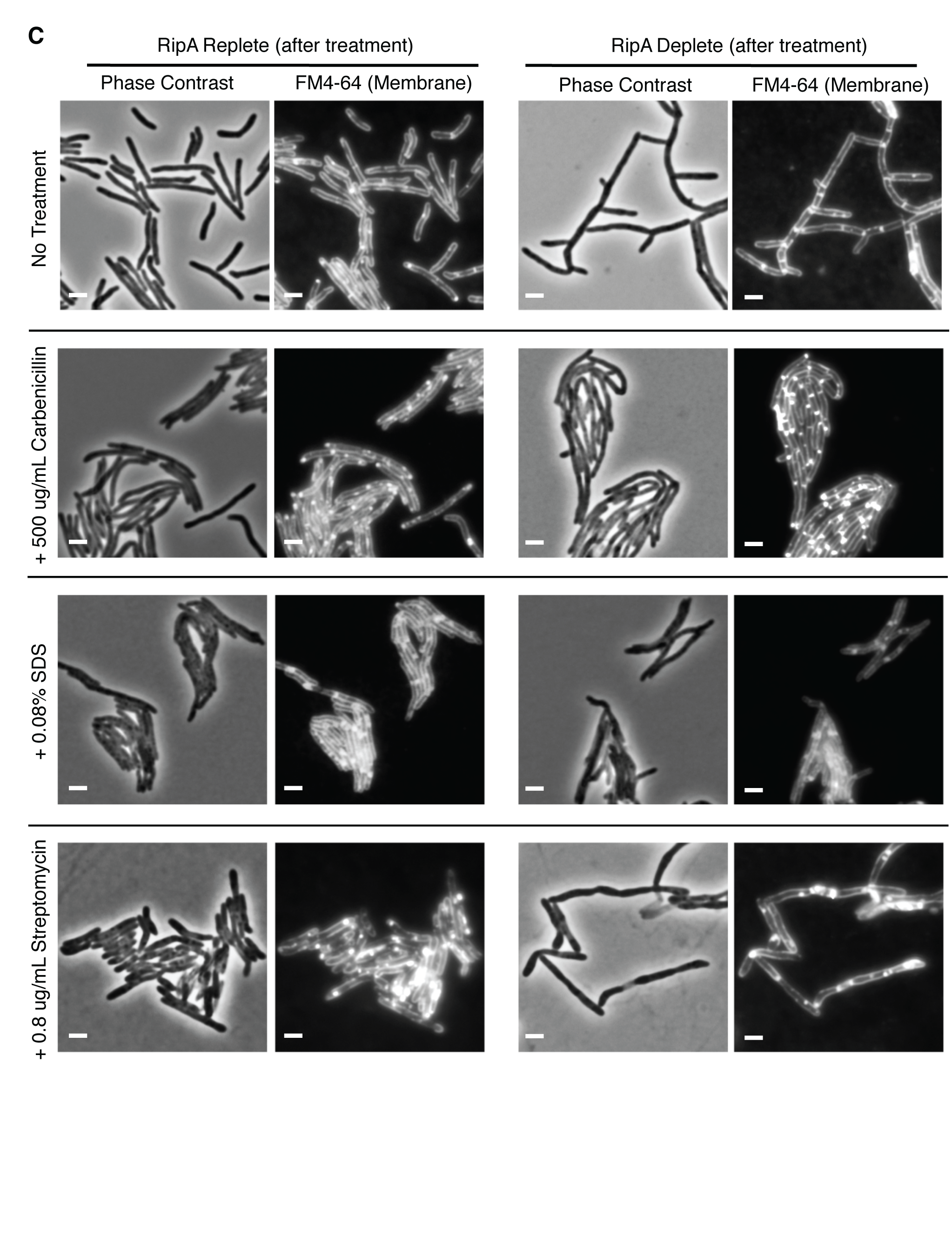

Supplement: Figure S2 — RipA depleted cells are not morphologically resistant to general stress. RipA replete and pre-depleted M. smegmatis were treated with various chemical stresses (0.8 µg/mL streptomycin, 0.08% SDS or 500 µg/mL carbenicillin for 6 hours, stained with FM4-64 and morphology assessed by fluorescent microscopy. Scale bar represents 2 µm. (TIF) [file ppat.1003197.s002.tif]

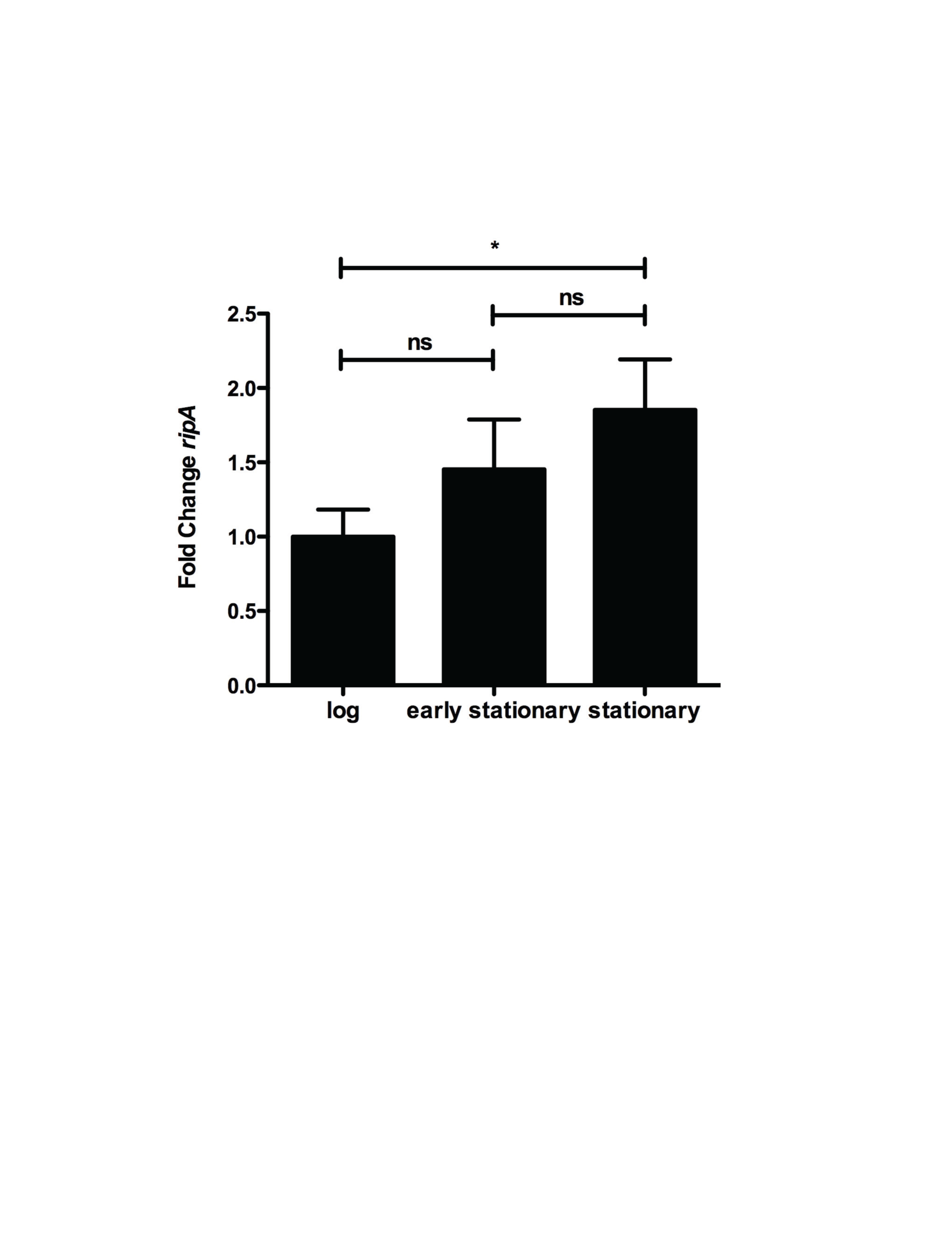

Supplement: Figure S3 — RipA is not post-transcriptionally downregulated. Quantitative PCR was performed against the ripA transcript during different growth phases of wildtype M. smegmatis. Expression was normalized to sigA transcripts in each sample. p-values: ns, non significant; *, p < 0.05. (TIF) [file ppat.1003197.s003.tif]

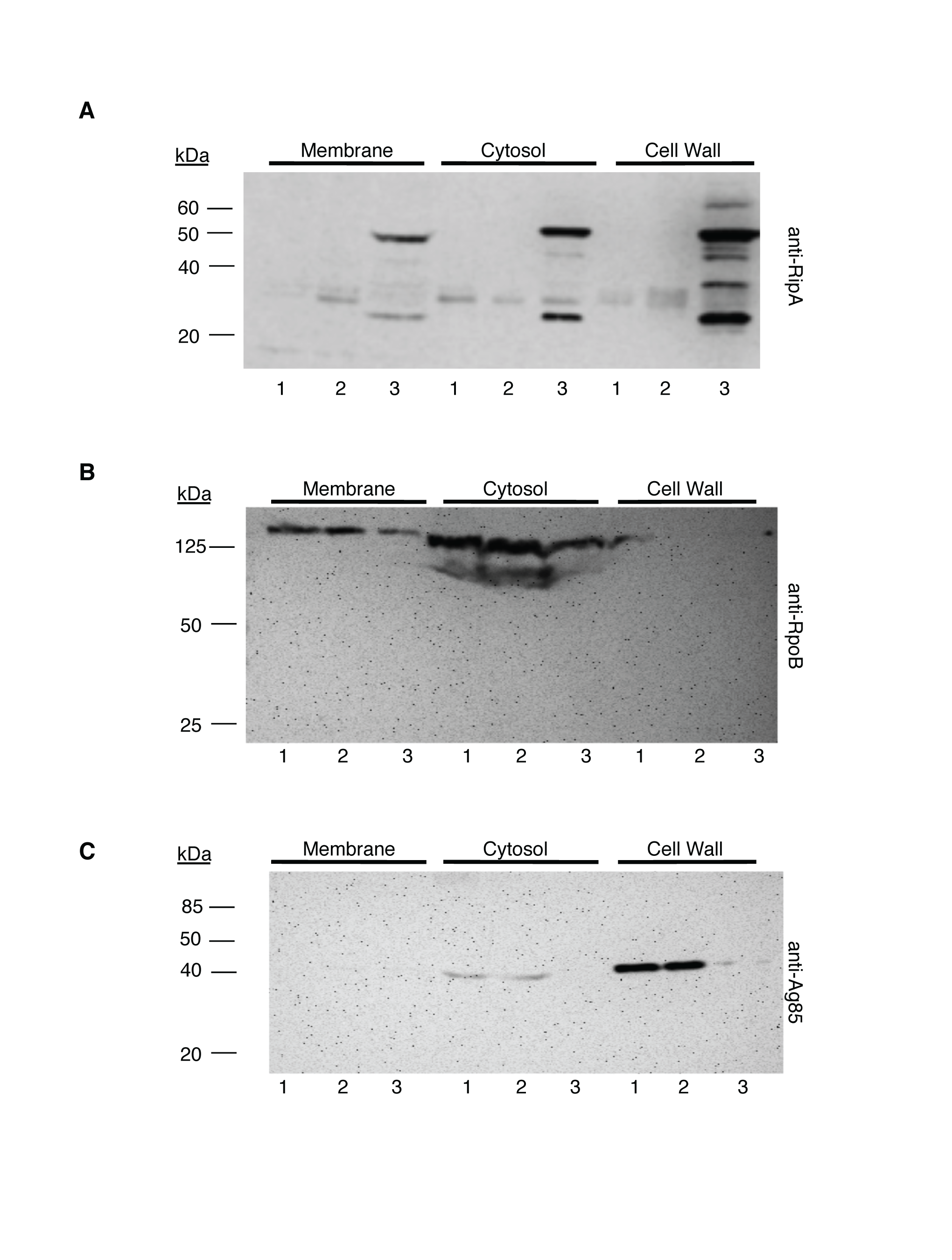

Supplement: Figure S4 — RipA processing is within the cell wall compartment. (A) Anti-RipA Western blot of fractionated wildtype M. smegmatis (lanes 2), cells depleted for RipA (lanes 1) or cells overexpressing inactive RipASm C408A (lanes 3). (B) Anti-RpoB Western blot of fractionated wildtype (lanes 1), RipA depleted (lanes 2) or RipASm C408A overexpressing (lanes 3) M. smegmatis. (C) Anti-Ag85 Western blot of fractionated wildtype (lanes 1), RipA depleted (lanes 2) or RipASm C408A overexpressing (lanes 3) M. smegmatis. Each lane was standardized to total protein from the cell wall fraction. Due to the heavy presence of recombinant RipA in RipASm C408A induced cells, upon protein normalization, there is an enrichment for RipA protein, which leads to an apparent decrease in other cell wall proteins, such as Ag85. (TIF) [file ppat.1003197.s004.tif]

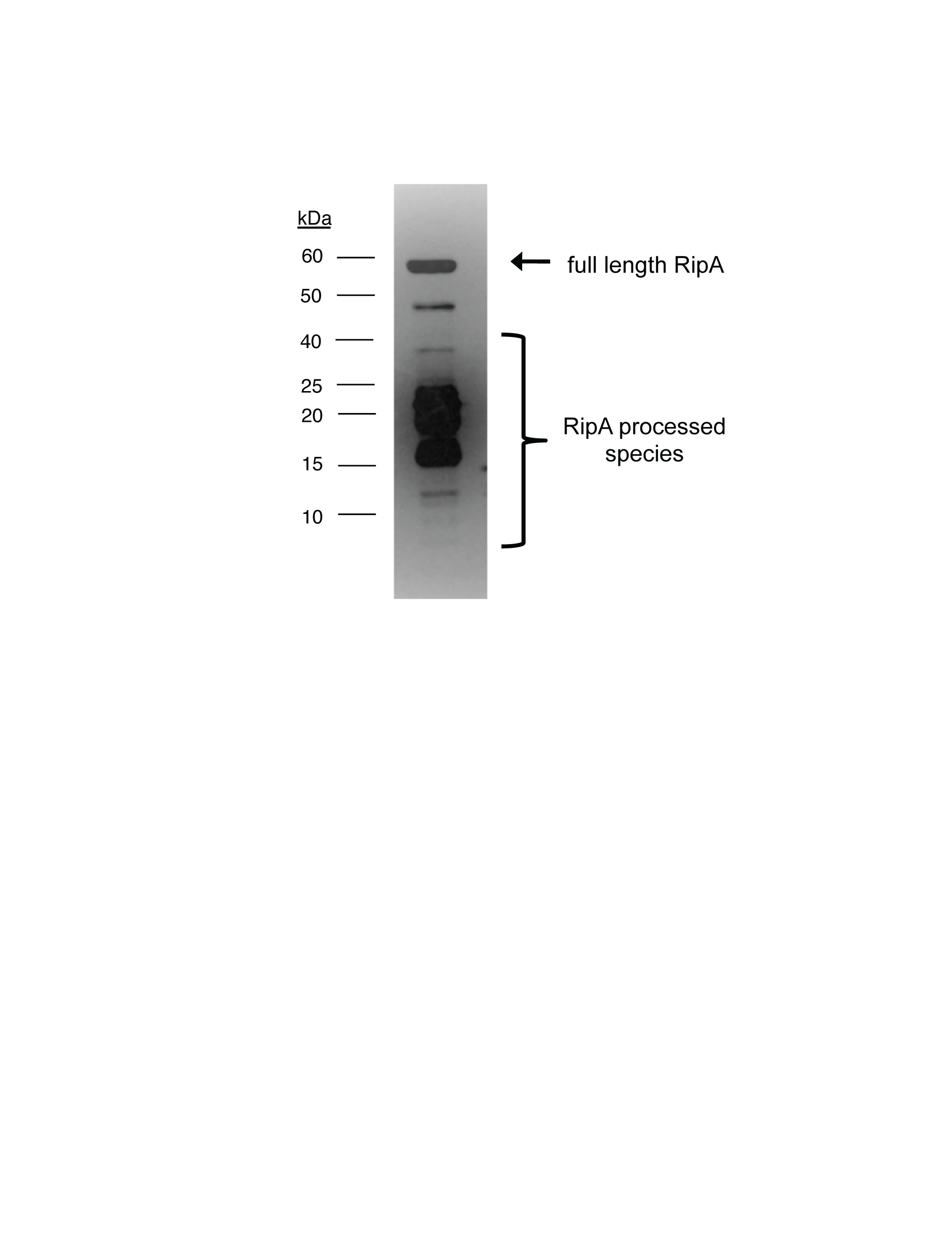

Supplement: Figure S5 — DP301 and DP315 residues are not necessary for RipA cleavage. Site-directed mutagenesis was used to create a RipASm DP300AA DP315AA double substitution mutant in the RipASm C408A background. This mutant (DP300AA DP315AA C408A) was fused to a FLAG tag and expression induced with aTc in M. smegmatis. Total cell lysate was run on SDS-PAGE and RipA processing was monitored by Western blot analysis using an anti-FLAG antibody. RipA processed species are indicated in brackets and RipA full length is indicated by an arrow. (TIF) [file ppat.1003197.s005.tif]

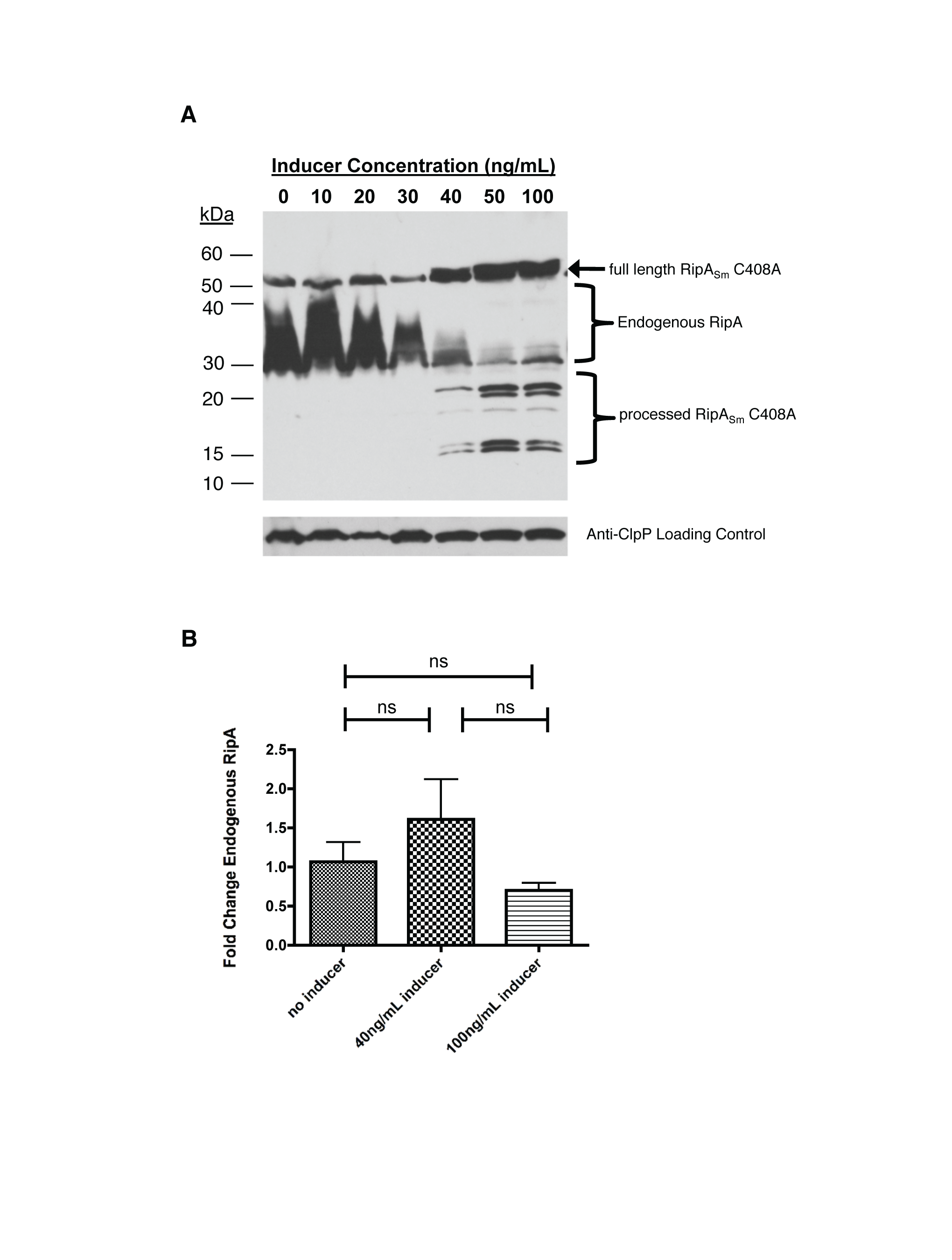

Supplement: Figure S6 — Overexpression of RipASm C408A is mild relative to endogenous RipA levels. (A) The M. smegmatis RipASm C408A overexpression strain was grown under various inducer concentrations and total protein harvested for Western blot analysis with anti-RipA antibody (top panel) and anti-ClpP antibody (loading control, bottom panel). Full length recombinant (arrow) and processed RipA C408A was detected (bottom brackets), as well as endogenous RipA (upper brackets). (B) Total RNA was harvested from cells grown in various concentrations of inducer and quantitative RT-PCR performed against endogenous RipA (primers detect the ripA 3′ UTR region) and normalized to sigA levels. ‘ns’ = not significant (p-value>0.1). (TIF) [file ppat.1003197.s006.tif]

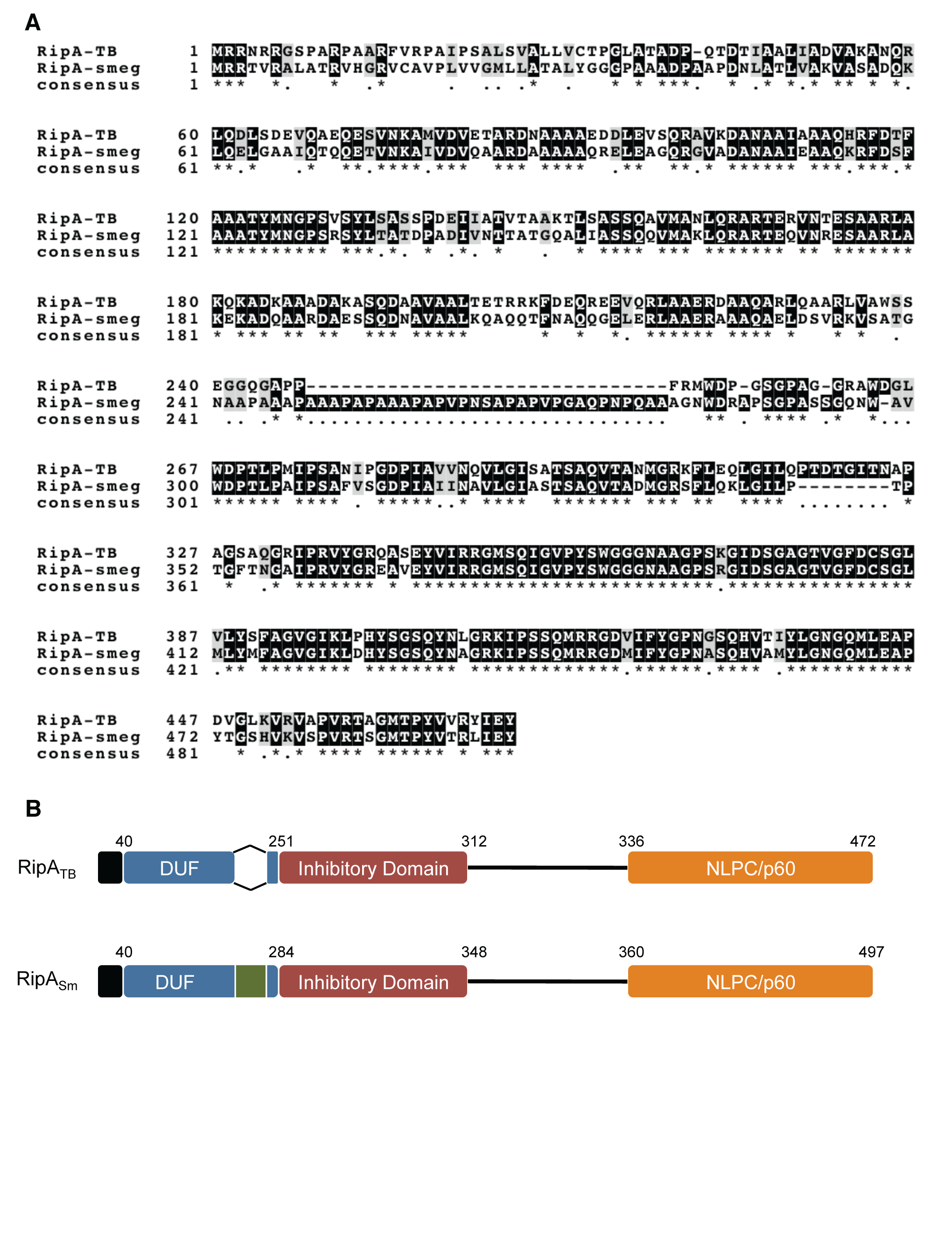

Supplement: Figure S7 — Alignments between RipA homologues from M. tuberculosis and M. smegmatis . (A) Amino acid alignment between RipA-TB from M. tuberculosis and RipA-smeg from M. smegmatis. Conserved residues are shown in black or denoted with ‘ * ’ in the consensus row, while shared amino acids with similar properties are in gray or marked with a ‘ . ’ in the consensus line. (B) Schematic diagram of the predicted domain structure of RipATB and RipASm from M. tuberculosis and M. smegmatis, respectively. Both proteins have an N terminal secretion signal (black), a DUF or domain of unknown function (blue), an inhibitory domain (red), an extended loop (black line) and an NLPC/p60 family peptidoglycan hydrolysis domain (orange). RipASm also has an additional 33 amino acid extension at the junction between the DUF and inhibitory domains (green box). (TIF) [file ppat.1003197.s007.tif]

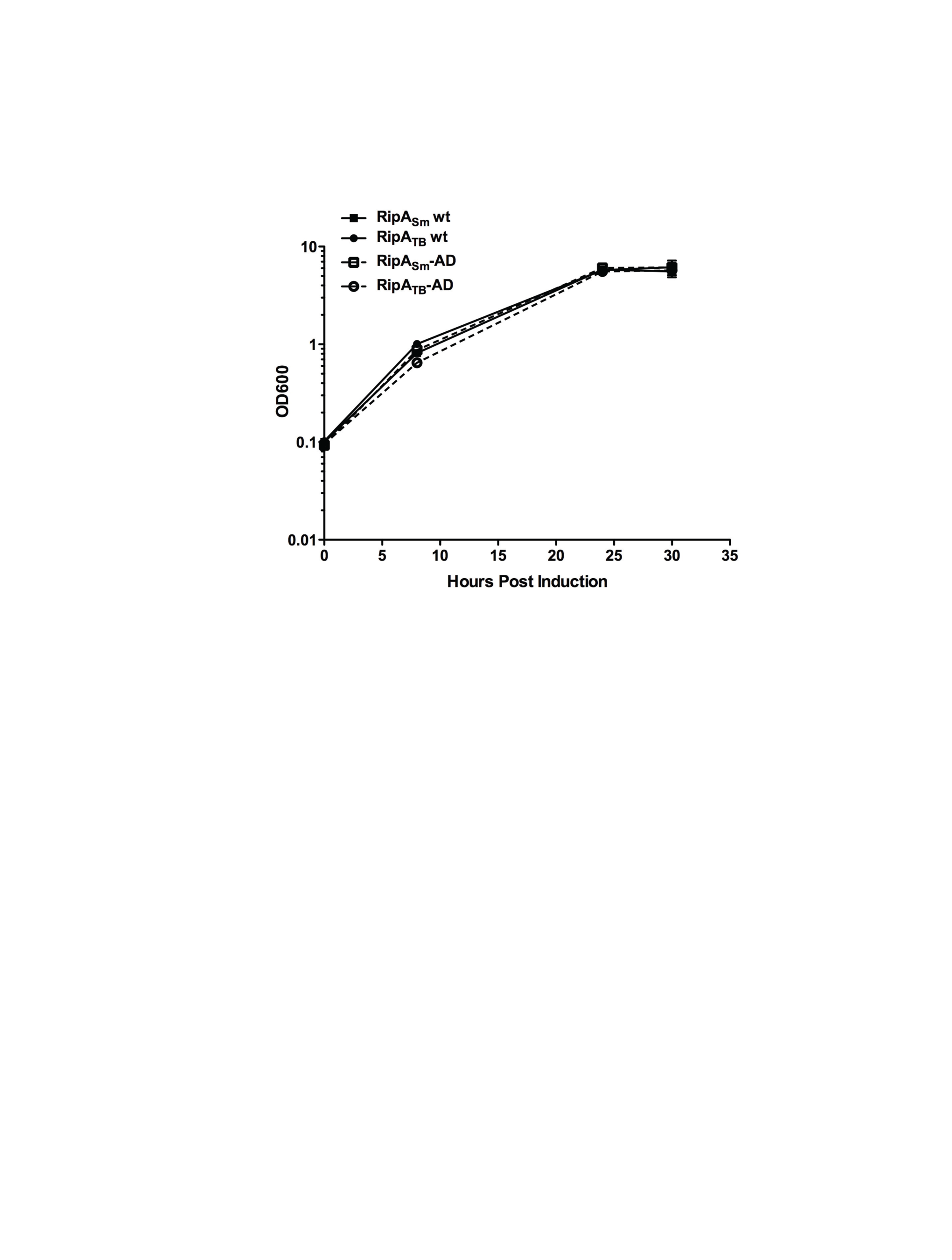

Supplement: Figure S8 — Uninduced strains of M. smegmatis were equally viable. M. smegmatis strains were constructed to overexpress full length RipATB and RipASm and truncated RipATB-AD, RipASm-AD constructs under the control of aTc. As negative controls, growth of these strains in the absence of inducer was assessed over time by OD600. (TIF) [file ppat.1003197.s008.tif]

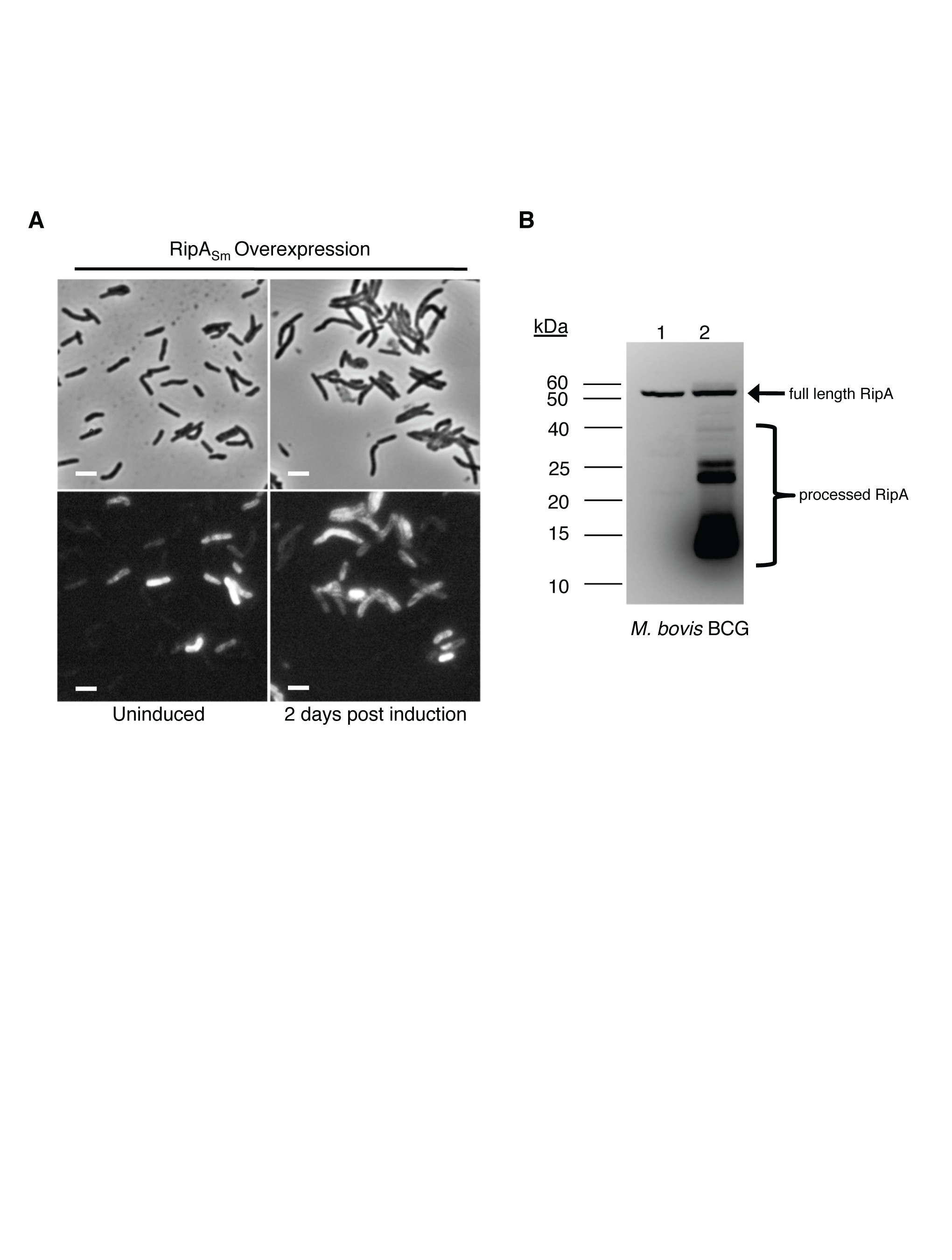

Supplement: Figure S9 — RipA processing is rate limiting in slow growing mycobacteria. (A) Full length RipASm was overexpressed in M. bovis BCG for 48 hours. Cells were analyzed for changes in morphology by microscopy. Membranes were stained with FM4-64. Scale bar represents 2 µm. (B) Anti-RipA Western blot of M. bovis BCG induced to overexpress RipASm (lane 2). Uninduced M. bovis BCG lysate was run as a control (lane 1). Full length RipA (arrow), as well as processed forms (brackets), were detected. (TIF) [file ppat.1003197.s009.tif]
